# Visualization and ligand-induced modulation of dopamine receptor dimerization at the single molecule level

**DOI:** 10.1038/srep33233

**Published:** 2016-09-12

**Authors:** Alina Tabor, Siegfried Weisenburger, Ashutosh Banerjee, Nirupam Purkayastha, Jonas M. Kaindl, Harald Hübner, Luxi Wei, Teja W. Grömer, Johannes Kornhuber, Nuska Tschammer, Nigel J. M. Birdsall, Gregory I. Mashanov, Vahid Sandoghdar, Peter Gmeiner

**Affiliations:** 1 Department of Chemistry and Pharmacy, Emil Fischer Center, Friedrich-Alexander University, Schuhstraße 19, 91052 Erlangen, Germany; 2 Max Planck Institute for the Science of Light and Department of Physics, Friedrich-Alexander University, Günther-Scharowsky-Straße 1/ Bldg. 24, 91058 Erlangen, Germany; 3 Department of Psychiatry and Psychotherapy, Friedrich-Alexander University, Schwabachanlage 6, 91054 Erlangen, Germany; 4 The Francis Crick Institute, Mill Hill Laboratory, Mill Hill, London NW7 1AA, UK

## Abstract

G protein–coupled receptors (GPCRs), including dopamine receptors, represent a group of important pharmacological targets. An increased formation of dopamine receptor D_2_ homodimers has been suggested to be associated with the pathophysiology of schizophrenia. Selective labeling and ligand-induced modulation of dimerization may therefore allow the investigation of the pathophysiological role of these dimers. Using TIRF microscopy at the single molecule level, transient formation of homodimers of dopamine receptors in the membrane of stably transfected CHO cells has been observed. The equilibrium between dimers and monomers was modulated by the binding of ligands; whereas antagonists showed a ratio that was identical to that of unliganded receptors, agonist-bound D_2_ receptor-ligand complexes resulted in an increase in dimerization. Addition of bivalent D_2_ receptor ligands also resulted in a large increase in D_2_ receptor dimers. A physical interaction between the protomers was confirmed using high resolution cryogenic localization microscopy, with ca. 9 nm between the centers of mass.

Class A G protein–coupled receptors (GPCRs) represent a large family of integral membrane proteins and major pharmacological targets^1^ which have traditionally been considered to exist and function as monomers. Biochemical and biophysical evidence has steadily accumulated indicating the ability of GPCRs to assemble as homodimers, heterodimers or higher-order oligomers^2^,^3^. A quantitative knowledge of the number and arrangement of protomers, the temporal dynamics of the interaction between monomers, dimers and higher-order oligomers, the effect of receptor ligands on these different conformations, and their pathophysiological roles are of particular interest^4^.

The development of resonance energy transfer (RET) based-techniques such as fluorescence and bioluminescence resonance transfer (FRET and BRET) have played an important role in the discovery and characterization of homo- and heteromers in living cells^2^,^5^,^6^,^7^,^8^. However, these techniques do not provide information about the degree and dynamics of di- and oligomerization at the single molecule level. Recent studies using single-molecule sensitive total internal reflection fluorescence microscopy (TIRF-M) allowed the visualization and tracking of individual GPCRs in the membrane of a living cell in real time^9^,^10^,^11^. Thus, the dynamics of muscarinic acetylcholine M_1_, M_2_ and N-formyl peptide receptors, their mobility and dimerization could be observed and quantified by using fluorescent ligands^9^,^10^,^12^. Related work utilized direct labeling of β_1_- and β_2_-adreneric receptors with rhodamine-type fluorophores via the SNAP-tag technology^11^,^13^. The studies revealed that dimerization of class A GPCRs at the plasma membrane can exhibit a transient equilibrium between dimers and monomers.

Dopamine D_2_-like GPCRs (D_2L,_ D_2S_ and D_3_) are associated with several central nervous system diseases including schizophrenia, Parkinson’s disease and drug addiction^14^. They offer, therefore, an essential and highly important set of drug targets^15^,^16^. Recent investigations indicate that D_2_-like receptors exist as homomeric^17^,^18^,^19^,^20^,^21^ or heteromeric complexes^20^,^22^ and an increased formation of D_2_ homodimers was suggested to be associated with the pathophysiology of schizophrenia^23^. Targeting of GPCR dimers and ligand-induced modulation of dimerization with selective chemical tools may allow the investigation of the signaling behavior of dimers and the pathophysiology of diseases that are potentially associated with GPCR dimerization. Such compounds may be bivalent ligands incorporating two pharmacophores connected by an appropriate linker that enables simultaneous binding to two adjacent receptor protomers^24^,^25^,^26^.

In this study, we applied TIRF-M to visualize individual fluorescently labeled dopamine D_2_-like receptors in the membrane of living CHO cells using either SNAP-tag technology or fluorescent ligands. This allowed us to study the spatial and temporal organization of the receptors at the single-molecule level under ligand-free and agonist- or antagonist-bound conditions. Furthermore, bivalent D_2_-like receptor antagonists^27^ were synthesized. We could show that these compounds are able to substantially shift the equilibrium between monomers and dimers toward D_2_ receptor dimers.

Moreover, we performed nanoscopic distance measurements in order to confirm a physical interaction between the two protomers of SNAP-tagged D_2L_ receptor dimers using cryogenic localization microscopy^28^,^29^. This super-resolution microscopy method has recently demonstrated both Angstrom precision and accuracy in resolving nanometer separations. The present study is the first adaptation of this technique to whole cells.

## Results

### Visualization and transient dimer formation of single SNAP-D_2L_ receptors in the membrane of living cells

**Figure 1:**
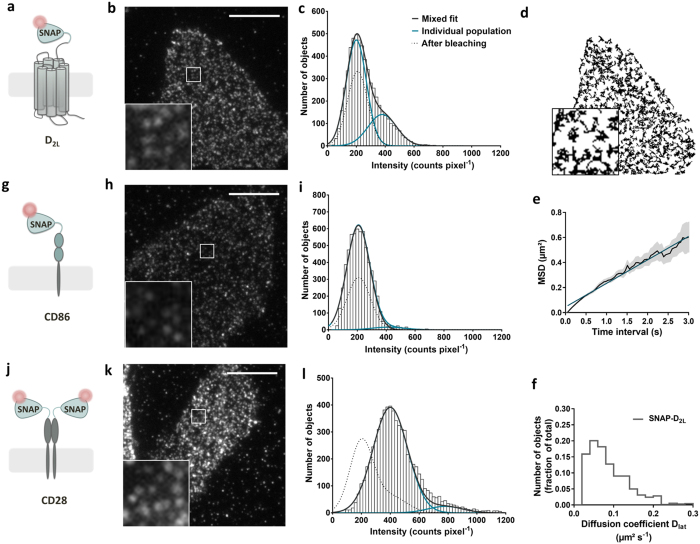
Visualization, tracking and analysis of the dimerization of single SNAP-tagged D_2L_ receptors using SNAP-CD86 and SNAP-CD28 as monomeric and dimeric reference proteins. (**a,g,j**) Schematic representation of the SNAP-tagged constructs. (**b,h,k**) Representative images of single CHO cells, stably transfected with the corresponding labeled protein and visualized by TIRF-M. Scale bar, 10 μm. The first 100 frames of the cell in b are shown in Supplementary Movie S1. Inserts correspond to higher magnification images of the areas in the white boxes. (**c,i,l**) Representative intensity distributions of fluorescent spots identified over the first 10-frame time window of TIRF illumination of CHO cells, stably transfected with the corresponding construct and labeled with Alexa546-BG. Number of identified particles, *n *= 5770 (**c**), 6252 (**f**) and 6458 (**i**). Data were fitted with a mixed Gaussian model. A mixed Gaussian fit after partial photobleaching (dotted line) was used to estimate the intensity of a single fluorescent molecules in each image sequence. (**d**) Individual trajectories of moving SNAP-D_2L_ receptors were identified from the entire recording of the cell shown in (**b)**. The insert shows a higher magnification that illustrates the random nature of the diffusive process. (**e**) Representative plot of the average mean square displacement (MSD) (mean ± s.d.) versus the time interval (δt) for the trajectories shown in (**a**). The plot is linear (r^2^ = 0.99 – linear fit (blue)), over a 3-s timescale, which is consistent with receptor movement following a random walk, and it shows no evidence for anomalous diffusive behavior. (**f**) Distribution of the diffusion coefficients of the receptor particles tracked in (**d**).

We used TIRF-M to visualize single dopamine receptors in the membrane of living cells. To investigate the spatial and temporal organization of receptor protomers under ligand-free conditions, we employed the SNAP-tag technology^13^. The dopamine D_2L_ receptor was *N*-terminally labeled with a SNAP-tag, which derives from the O^6^-guanine nucleotide alkyltransferase (ATG). The fusion protein was reacted with a fluorescent benzyl guanine (BG) allowing covalent labeling (Fig. 1a). By investigating cAMP accumulation, the SNAP-D_2L_ receptor was shown to be functionally identical to wild-type D_2L_ receptors (Supplementary Fig. S1f). CHO cells stably expressing the SNAP-D_2L_ receptor were generated with an expression level of 1000 ± 30 fmol mg^−1^ protein (mean ± s.e.m.), which is in the range of endogenous expression levels of dopamine receptors *in vivo*^30^.

To visualize SNAP-D_2L_ receptors in the membrane of stably transfected CHO cells, the cells were specifically labeled with saturating concentrations of Alexa546-BG and imaged by TIRF-M (Supplementary Fig. S1). SNAP-D_2L_ receptors could be identified as individual fluorescent spots moving rapidly in the plasma membrane with a density of 0.67 ± 0.03 spots μm^−2^ (mean ± s.d., 10 cells), and they were automatically detected and tracked (Fig. 1a,b,d, Supplementary Movie S1). Analysis of the individual receptor spatial trajectories allowed us to calculate their mean squared displacement (MSD) over a range of time intervals (δt) and, hence, to characterize the receptor motion. The linear relationship between MSD and δt showed that receptor diffusion is consistent with a simple Brownian random walk, which is characteristic of free diffusion in the membrane (Fig. 1e). There was no evidence of restricted or anomalous diffusion over the timescale explored in this study (50 ms–3 s). The calculation of the diffusion coefficient from MSD data revealed an average diffusion coefficient (D_lat_) of 0.084 ± 0.006 μm^2^ s^−1^ (mean ± s.d., 12031 tracks, 10 cells) for labeled SNAP-D_2L_ receptors, which is similar to the values measured for M_1_^9^, β_1_ and β_2_^11^ receptors (Fig. 1f, Supplementary Table S2). We analyzed the intensity distribution of fluorescent spots observed at the beginning of the video recording (Fig. 1c). The histogram generated from the data was skewed due to the presence of two underlying populations and it was fitted by the sum of two Gaussian functions to estimate the fractions of these populations. The predominant peak (70%) at 197 ± 80 counts pixel^−1^ (mean ± s.d.) corresponded to the intensity of single Alexa546-BG molecules on glass slides (Supplementary Note S1, Supplementary Fig. S2) and could be attributed to receptor monomers. The second population (30%, intensity 400 ± 110 counts pixel^−1^ (mean ± s.d.)), corresponding to double the intensity of the monomer, and represented receptor dimers. These results are similar to those reported for the M_1_ and β_1_ receptors^9^,^11^.

As controls for the specificity of receptor dimerization observed with the SNAP-D_2L_ receptors and for our labeling, tracking and analysis methods, we used CD86 and CD28 as monomeric and dimeric reference proteins fused with a SNAP-tag at the extracellular *N*-terminus (SNAP-CD86 and SNAP-CD28) (Fig. 1g,j). We generated stably transfected CHO cell lines of both control constructs which, after labeling with Alexa546-BG and TIRF-illumination, revealed equivalent fluorescent spot densities compared to the stably expressing SNAP-D_2L_ cell line (Supplementary Table S3). Intensity distribution analysis of the fluorescent spots of SNAP-CD86, (Fig. 1g–i), gave a predominant peak with an average intensity of 200 ± 80 counts pixel^−1^ (mean ± s.d.) and revealed that ≥95% were monomeric, with the remaining existing probably as randomly colocalized spots at a distance below the resolution limit of the system. This provided comfort that the dimer fraction of 30% produced at these levels of expression of SNAP-D_2L_ receptors did indeed reflect a true receptor interaction. To examine whether this approach could detect constitutive dimers, we studied the disulfide-bridged protein dimer SNAP-CD28 (Fig. 1j–l). From the intensity distribution shown in Fig. 1l, it was concluded that 90% of the detected fluorescent spots represent dimers with an intensity of 400 ± 110 counts pixel^−1^ (mean ± s.d.) and twice that measured with the monomeric SNAP-CD86. Both control proteins could be tracked with the same accuracy compared to the SNAP-D_2L_ receptor, as determined by calculating the mean trajectory lifetime of each construct which showed no significant differences (Supplementary Fig. S3a,b). These results validated the analytical approach and confirmed the specificity of SNAP-D_2L_ receptor dimerization.

**Figure 2:**
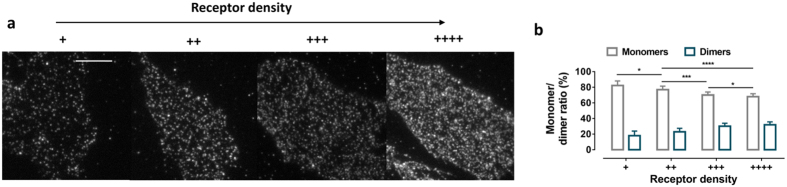
Dependence of the distribution of monomers and dimers on receptor density. (**a**) Representative receptor density level: **+** 0.38 ± 0.016 spots μm^−2^ (mean ± s.d., 8 cells), **++** 0.52 ± 0.039 spots μm^−2^ (mean ± s.d., 10 cells), **+++** 0.67 ± 0.031 spots μm^−2^ (mean ± s.d., 10 cells), **++++** 0.79 ± 0.046 spots μm^2^ (mean ± s.d., 10 cells). (**b**) The monomer/dimer ratios (mean ± s.d.) were calculated from the fitted fluorescence intensity distributions of fluorescent ligand receptor complexes using a mixed Gaussian model (compare to Fig. 1c). The statistical significance of the differences in monomer levels in the four groups was determined by an unpaired t-test (*p-value < 0.05, ***p-value < 0.010, ****p-value < 0.0001).

Investigation of cell lines with reduced expression of the SNAP-D_2L_ receptor showed a reduced fraction of dimers (Fig. 2). A density dependent behavior has also been described for β_1_- and β_2_-adreneric receptors^11^ and is expected for reversible dimerization.

**Figure 3:**
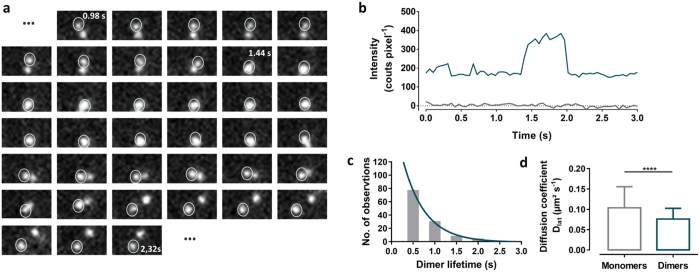
Transient dimer formation of SNAP-D_2L_ receptors. (**a**) 48 sequential frames of two Alexa546-labeled SNAP-D_2L_ receptors showing transient dimer formation (frame rate of 19.32 fps) (also shown in Supplementary Movie S3). (**b**) Intensity profile (blue) of the marked fluorescent SNAP-D_2L_ receptor shown in (**a)**, compared to background intensity (grey). (**c**) A histogram of the lifetimes of 120 SNAP-D_2L_ receptor dimers taken from trajectories similar to those in (**a)** and collected in 0.5 s bins. The solid line represents a one-phase exponential fit for a mean lifetime of 0.50 s (95% confidence interval: 0.44–0.60). (**d**) Effect of the size of SNAP-D_2L_ receptors on their lateral diffusion. The diffusion coefficients (D_lat_) of the analyzed receptor particles (*n*) are shown (monomers −0.104 ± 0.052 μm^2^ s^−1^, *n* = 412 from 3 cells and dimers −0.075 ± 0.027 μm^2^ s^−1^, *n* = 373 from 3 cells. Data represent mean ± s.d. The difference, determined by an unpaired t-test (****p-value < 0.0001) is significant and shows that the receptor mobility is negatively correlated with the size of the receptor complexes.

To address the dynamic behavior of SNAP-D_2L_ receptors dimerization, the fluorescence intensities of individual trajectories were visually inspected (Fig. 3a, Supplementary Movie S3). As illustrated in Fig. 3b, we found that intensity doubling was associated with dimer formation within a trajectory arising from two monomers, forming a dimer, followed by the dissociation of the dimer into two monomers. From an analysis of the lifetimes of 120 dimers with trajectories similar to that shown in Fig. 3b, the lifetime of the transient formation of dimers could be shown to be approximately 0.50 s (95% confidence interval: 0.44–0.60) (half-life (t_1/2_) of 0.35 s time (95% confidence interval: 0.31–0.41) (Fig. 3c). We also observed that the rate of receptor diffusion was negatively correlated with the size of receptor complexes (Fig. 3d). As expected, larger dimers diffused more slowly (0.075 ± 0.027 μm^2^ s^−1^, *n* = 373 from 3 cells, mean ± s.d.) compared to the smaller monomers (0.104 ± 0.052 μm^2^ s^−1^, *n* = 412 from 3 cells, mean ± s.d.)^31^, a diagnostic behavior that could be also shown for the monomer and dimer control proteins SNAP-CD86 and SNAP-CD28, respectively (Supplementary Fig. S3d, Supplementary Table S3).

### Cryogenic localization microscopy for nanometer distance measurement of two protomers in a SNAP-tagged D_2L_ receptor dimer

**Figure 4:**
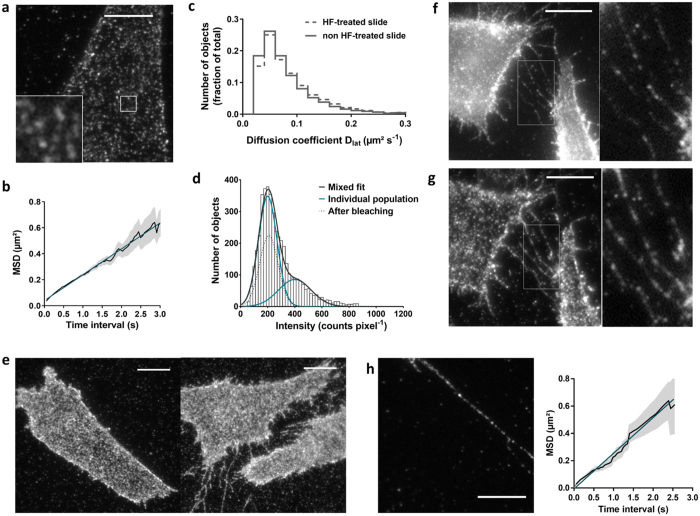
Visualization, diffusion and dimerization of Alexa546-labeled SNAP-D_2L_ receptors and on membrane protrusions using HF-treated slides. (**a**) Representative images of a single CHO cell stably expressing the SNAP-D_2L_ receptor and seeded on HF-treated glass slides, labeled with Alexa546-BG and visualized by TIRF-M. The insert corresponds to higher magnification image of the area in the small white box. (**b**) Plot of mean square displacement (MSD ± s.d.) versus the time interval (δt) of receptor particles that were tracked in a. The plot is linear (r^2^ = 0.99 – linear fit (blue)), over a 3-s timescale, which is consistent with receptor movement following a random walk. (**c**) The distribution of the diffusion coefficients of the analyzed receptor particles (*n*) are shown (*n* = 4409, 5 cells - HF-treated slide; *n* = 4409, 8 cells - non HF-treated slide) and revealed no evidence for anomalous diffusive behavior as a result of HF-treatment. (**d**) Representative intensity distribution of fluorescent spots identified over the first 10-frame time window of TIRF-illumination. Data were fitted with a mixed Gaussian model (sum of two Gaussian functions). (**e**) Representative TIRF-M images of CHO cells stably transfected with SNAP-D_2L_ receptor, incubated in iso-osmotic (300 mOsm) (left) and hypo-osmotic PBS (108 mOsm) (right) for 2 h and labeled with Alexa546-BG. (**f,g**) Imaging of a region of membrane protrusions of CHO cells stably transfected with the SNAP-D_2L_ receptor, incubated in hypo-osmotic (108 mOsm) PBS for 2 h and labeled with Alexa546-BG in epi-illumination (**f**) and TIRF-illumination (**g**). (**h**) Representative images of one membrane protrusion of a CHO cell stably expressing the labeled SNAP-D_2L_ receptor and visualized by TIRF-M. (**f**) Plot of mean square displacement (MSD ± s.d.) versus the time interval (δt) of receptor particles that were tracked in (**h**). The plot is linear (r^2^ = 0.99 – linear fit (blue)), over a 2,5-s time scale and the calculation of the average diffusion coefficient D_lat_ of 0.077 ± 0.007 μm^2^ s^−1^ (mean ± s.d., 16 regions of membrane protrusions of 8 cells) revealed no evidence for anomalous diffusive behavior in the membrane protrusions. Scale bars, 10 μm.

To investigate the physical interaction of two dopamine receptor protomers in a dimer configuration, we applied cryogenic localization microscopy as a super-resolution technique, where the stochastic blinking behavior of the fluorophores was used to distinguish and localize them individually. The method allows nanometer distance measurement with angstrom accuracy^28^,^29^. We prepared samples with CHO cells stably expressing SNAP-D_2L_ receptors adhered to clean fused silica cover slides. To increase the adherence of the cells, the cover slides were treated with hydrofluoric (HF) acid prior to a thorough cleaning procedure. We verified that the HF treatment did not influence the diffusion and dimerization of SNAP-D_2L_ receptors (Fig. 4a–d). The samples were then mounted in the vacuum chamber of a flow cryostat and cooled to liquid helium temperature (T = 4.3 K). In this experiment (Supplementary Fig. S4 for a schematic drawing of the optical and cryogenic setup), the microscope objective was located outside of the vacuum chamber so that objective-based TIR-illumination was not accessible. Instead, we used a wide-field epi-illumination, which was accompanied by a higher background level caused by out-of-focus fluorescence compared to samples fabricated by spin-coating extremely clean solutions of purified biomolecules. Because of the larger absorption cross-sections of chromophores at cryogenic temperatures this effect is particularly dramatic in our experiment. To address this issue, we reduced the amount of cellular material in the region of interest without affecting the SNAP-D_2L_ receptor diffusion by inducing filopodia tube-like membrane protrusions through exposure of the cells to hypo-osmotic stress conditions (Fig. 4e,h). The direct comparison between wide-field epi-illumination and TIRF-mode for the same CHO cell at room temperature showed that the level of background fluorescence in the membrane protrusion regions is similar (Fig. 4f,g). We thus concentrated our analysis on these regions during the cryogenic measurements. There have been several attempts to deal with higher labeling densities and the resulting overlapping fluorophores, e.g. super-resolution optical fluctuation imaging (SOFI)^32^,^33^ and the Bayesian localization analysis (Bayesian Bleaching and Blinking, 3B analysis)^34^. The latter models the complete data set taking into account the number of fluorophores, their positions and their on/off states, and performs a global optimization. This way, blinking fluorophores that reappear at a later, not necessarily adjacent, frame will have an improved localization precision.

**Figure 5:**
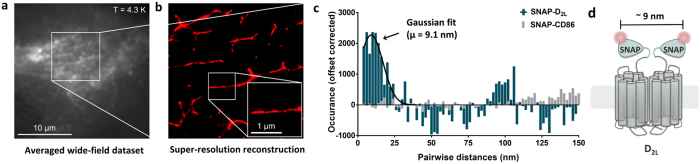
Cryogenic localization microscopy of SNAP-D_2L_ receptor dimers. (**a**) Averaged wide-field dataset of a recording of a CHO cell stably expressing labeled SNAP-D_2L_. (**b**) Super-resolution reconstruction after 3B analysis of the area indicated by the white square in (**a)**. (**c**) Histogram of the pairwise distances calculated from the emitter positions of the SNAP-D_2L_ (blue) and SNAP-CD86 monomers (gray). The Gaussian fit determines the separation of the SNAP-D_2L_ protomers to be μ = 9.1 ± 11.3 nm. (**d**) Schematic representation of an Alexa564-labeled SNAP-D_2L_ receptor dimer detected by cryogenic localization microscopy.

We decided to apply the 3B analysis to our data that were recorded at cryogenic temperatures. Figure 5a shows the time-averaged recording of two CHO cells at cryogenic temperatures. A region with membrane protrusions is located between the two cells (white square). Applying the 3B analysis to this region yielded the super-resolution reconstruction image shown in Fig. 5b. The localization precision was determined to be better than 10 nm by analyzing the widths of cross-sections. To extract distance information from our experimental data, we computed pairwise distances from the positions of the fluorophores as determined by the 3B analysis. Figure 5c shows a histogram of the pairwise distances for the SNAP-D_2L_ dopamine receptor (blue). The histogram is corrected by subtracting a simulated histograms which takes into account both an offset caused by random distances due to either background fluorescence mistakenly identified as fluorophores or receptors in a monomeric state as well as the geometry of the membrane protrusions (see Methods). As a control experiment, we looked at CHO cells stably expressing SNAP-CD86 monomers (grey histogram in Fig. 5c). There is a clear peak in the histogram for SNAP-D_2L_ (blue) at 9.1 ± 11.3 nm (mean ± s.d.) which is not present in the control experiment (grey). The experimentally obtained average distance was thus about 9 nm (Fig. 5d) and is in good agreement with the range of distances between two Alexa546 fluorophores (approximately 3–13 nm) provided from a homology model (Supplementary Fig. S5, Supplementary Note S2).

### Specific receptor ligands are able to modulate the monomer-dimer ratio

**Figure 6:**
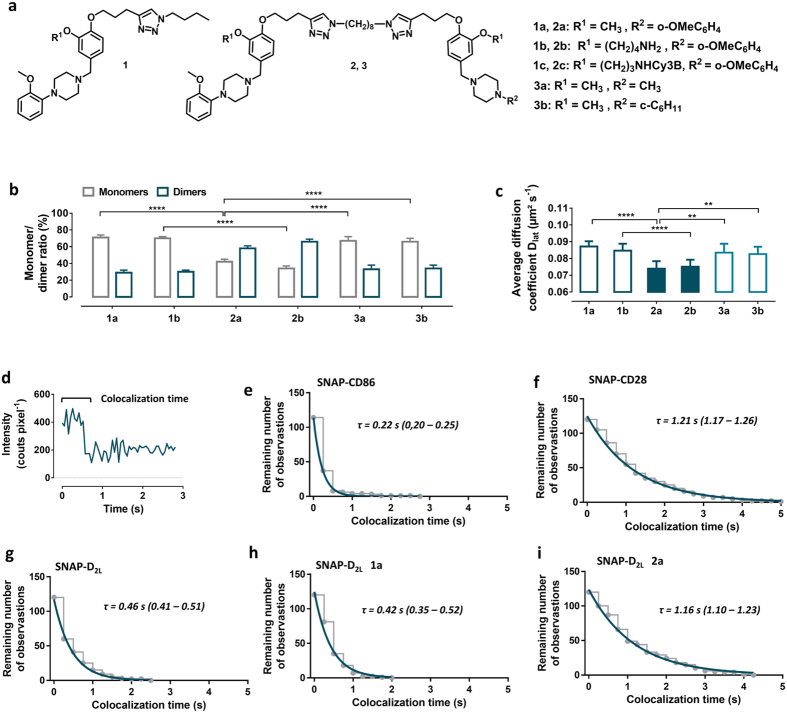
Influence of monovalent (1a,b), bivalent (2a,b) and bivalent control (3a,b) dopamine D_2_ receptor antagonists on receptor dimerization. (**a**) Chemical structures of monovalent ligands (**1a–c**), bivalent ligands (**2a–c**), and control ligands (**3a,b**). (**b**) Monomer/dimer ratios calculated from fitted fluorescence intensity distributions of Alexa546-labeled SNAP-D_2L_ receptors incubated with monovalent (**1a,b**), bivalent (**2a,b**), control (**3a,b**) ligands using a mixed Gaussian model (Supplementary Table S2, Supplementary Fig. S6a). Data represent mean ± s.d. of *n* analysed cells (*n* = 16 for **1a**, 8 for **1b**, 8 for **2a**, 16 for **2b**, 6 for **3a** and 8 for **3b**. (**c**) Average diffusion coefficients (D_lat_) of the corresponding ligand-SNAP-D_2L_ receptor complexes of the same analyzed cells in (**b**). Data in (**b,c**) represent mean ± s.d., Statistical analysis was performed by an unpaired *t*-test (**p-value < 0.01, ****p-value < 0.0001) and showed that the receptor mobility is negatively correlated with the size of the receptor complexes. (**d–g**) Comparison of the apparent lifetimes of particle colocalization of the monomeric SNAP-CD86 and dimeric SNAP-CD28 control proteins proteins (**e** and **f** respectively) and the SNAP-D_2L_ receptor in the absence and presence of the monovalent ligand **1a** or bivalent ligand **2a** (**g, h**, and **f** respectively). (**d**) Representative intensity profile of one trajectory which showed intensity doubling from the beginning of the particle tracking followed by one step intensity change which was used to calculate the colocalization time of two particles. (**e–i**) The apparent lifetime of particle colocalizations (τ; 95 confidence interval) was calculated by fitting colocalization time data with a one-phase exponential decay function. 120 trajectories like those shown in a were analyzed from 8 different cells in (**e**), 4 in (**f**), 6 in (**g**), 6 in (**h**) and 4 in (**i**), respectively.

**Figure 7:**
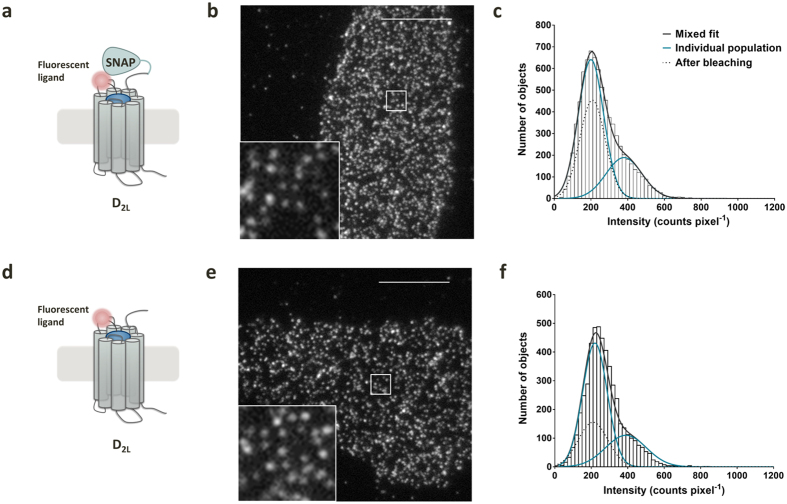
Visualization, tracking and analysis of the dimerization of single SNAP-tagged D_2L_ receptors and wild-type D_2L_ receptors labeled with the fluorescent antagonist 1c. (**a,d**) Schematic representation of the constructs. (**b,e**) Representative images of single CHO cells stably transfected with the two constructs, labeled and visualized by TIRF-M. Spot densities were 0.69 spots μm^−2^ (**b**) and 0.64 spots μm^−2^ (**e**). Inserts correspond to higher magnification images of the areas in the white boxes. Scale bar, 10 μm. (**c,f**) Representative intensity distributions of fluorescent spots identified over the first 10-frame time window of TIRF illumination of CHO cells, stably transfected with the corresponding constructs and labeled with the fluorescent ligand **1c** (300 nM). Data were fitted with a mixed Gaussian model. A mixed Gaussian fit after partial photobleaching (dotted line) was used to estimate precisely the intensity of a single fluorescent spot in each image sequence.

We investigated whether the ratio between monomers and dimers could be modulated by specific dopamine receptor ligands. Chemical synthesis of the fluorescently labeled bivalent ligands of type **2** and monovalent agonist and antagonists of types **1**, **4** and **5** was achieved starting from the respective pharmacophores that were substituted with an alkyne-functionalized handle (Figs 6a and 7a, Supplementary Fig. S11–S16). Using click chemistry, linker units were introduced by copper-catalyzed azide–alkyne cycloaddition (CuAAC)^35^. The primary amino groups of the generated triazoles were coupled to Cy3B-NHS ester (which was used because of its excellent photophysical properties) to form the respective ligand-fluorophore conjugates. Non-fluorescent analogs and further control ligands were synthesized employing the same strategy.

#### Bivalent Ligands

CHO cells stably expressing the SNAP-D_2L_ receptor were incubated with the dopamine receptor antagonist **1a**, incorporating the privileged 1,4- disubstituted aromatic piperazine structure (1,4-DAP)^36^,^37^,^38^, or its bivalent analogue **2a**, comprising of two pharmacophores calculated of being capable of bridging adjacent binding sites of physically interacting dimers (Fig. 6a)^27^. In parallel, we synthesized the analogues **1b** and **2b** containing a hydrophilic substituent to improve aqueous solubility (Fig. 6a, Supplementary Figs S11 and S12). A concentration of 10-fold of the K_i_ value (Supplementary Table S1), which gives 91% receptor occupancy, was used as the labeling concentration for the monovalent and bivalent ligands **1a-c, 2a-c** and **3a,b.** We observed that the antagonists **1a** and **1b** had no effect on the monomer/dimer ratio nor on the mobility of the SNAP-D_2L_ receptor compared to the ligand-free conditions (Fig. 6b, Supplementary Fig. S6a, Supplementary Table S2). In contrast, the bivalent antagonists **2a** and **2b** increased the fraction of dimers from 30% to 66% and 58%, respectively. The higher extent of dimerization was also reflected in a significantly reduced mobility of the receptor bivalent ligand complexes with an average diffusion coefficient of 0.074 ± 0.004 μm^2^ s^−1^ for **2a** and 0.075 ± 0.004 μm^2^ s^−1^ (mean ± s.d.) for **2b** compared to 0.087 ± 0.003 μm^2^ s^−1^ and 0.085 ± 0.004 μm^2^ s^−1^ (mean ± s.d.) for receptor monovalent ligand complexes **1a** and **1b**, respectively (Fig. 6c, Supplementary Fig. S6b, Supplementary Table S2). T To confirm a receptor chelating binding mode of the bivalent ligands **2a** and **2b**, a comparative analysis using unsymmetrically substituted analogues^27^ that are composed of one pharmacophore which is connected via a spacer to structurally similar non-binding motif with a methyl **3a** or cyclohexyl **3b** unit displacing the methoxyphenyl head group was undertaken (Fig. 6a). The observed monomer/dimer ratios (67%: 33%, **3a** and 66%: 34%, **3b**) and average diffusion coefficients (0.083 ± 0.004 μm^2^ s^−1^ for **3a** and 0.083 ± 0.005 μm^2^ s^−1^ for **3b**, (mean ± s.d.)) were similar to those observed for the monovalent ligands **1a** and **1b** (Fig. 6b,c).

The results indicate, that the two D_2_ binding pharmacophores of the bivalent ligands **2a** and **2b** are essential and confirm a bivalent, receptor-bridging binding mode, which was also observed in cell lines with a lower SNAP-D_2L_ expression level (0.52 ± 0.039 μm^2^ s^−1^, mean ± s.d.) (Supplementary Fig. S6c). An increase of the labeling concentration to 100-fold of the K_i_ value of the monovalent and bivalent ligands, **1a,b** and **2a,b** respectively, did not have any further effects on SNAP-D_2L_ receptor dimerization and mobility (Supplementary Table S2).

The receptor bridging binding mode of the bivalent ligand **2a** was further verified by the determination of the colocalization time of the two receptors in a dimer. Using SNAP-D_2L_ receptor expressing cells, we recorded the time period from the beginning of the image acquisition and particle tracking in the presence and absence of the monovalent **1a** and bivalent **2a** ligands. The data were compared to those obtained from the monomeric and dimeric SNAP-control constructs (Fig. 6d-i). The observed mean apparent colocalization lifetime, *τ*. obtained by fitting the data to an exponential decay was 1.16 s (95% confidence interval: 1.10–1.23) which is almost identical to that of the dimeric control protein SNAP-CD28 1.21 s (95% confidence interval: 1.17–1.26). The *τ*-values for the ligand free SNAP-D_2L_ receptor and for the SNAP-D_2L_ receptor incubated with the monovalent ligand **1a** were estimated to be 0.46 s (95% confidence interval: 0.41–0.51) and 0.42 s (95% confidence interval: 0.35–0.52), respectively, which differs from the *τ*-value for the monomeric control protein SNAP-CD86 of 0.22 s (95% confidence interval: 0.20–0.25) which is associated with random colocalization of protein molecules. The data are thus compatible with the receptor dimer being stabilized by the bivalent ligand **2a** during the complete image acquisition time.

To estimate the lifetime of the receptor binding mode of the bivalent ligand, we extended our approach by labelling the D_2L_ receptor with the monovalent and bivalent fluorescent ligands **1c** and **2c.** This allowed us to measure the dissociation kinetics of the ligand by direct live cell dissociation experiments (for single molecule characterization of the fluorescent monovalent ligand **1a** and bivalent ligand **2b**, see Supplementary Note S3, Supplementary Fig. S7).

CHO cells stably expressing the SNAP-D_2L_ receptor were preincubated for 60 min with the fluorescent monovalent ligand **1c** (Supplementary Fig. S8a) or the bivalent ligand **2c** with a labeling concentration of 10-fold K_i_. rapidly washed and further incubated for different periods of time in the presence of 5 × 10^−6 ^M spiperone. As depicted in Supplementary Fig. S8b, the binding of both fluorescent ligands decreased exponentially. The monovalent ligand **1c** dissociated with half-life time t_1/2_ of 19.2 ± 0.6 min (mean ± s.e.m) (*τ* = 27.6 min (95% confidence interval: 23.6–31.6 min) whereas the bivalent ligand **2c** dissociated two fold slower with a t_1/2_ of 38.2 ± 2.0 min (mean ± s.e.m) (*τ* = 55.2 min (95% confidence interval: 47.6–62.7). These results indicate a very slow dissociation rate for type **1** and type **2** ligands.

The dissociation curves verified that that during the image acquisition the dissociation of the monovalent and bivalent ligands was negligibly small, which otherwise could have affected the estimates of the monomer/dimer ratios.

Using fluorescent ligands enabled us to monitor non-tagged D_2L_ receptor molecules. A CHO cell line stably expressing D_2L_ receptors at 1041 ± 15 fmol mg^−1^ protein (mean ± s.e.m.), a level which is similar to the SNAP-D_2_ _L_ cell line, was used. Labeling of the SNAP-D_2L_ and wild type D_2L_ receptor with the monovalent fluorescent ligand **1c** at a concentration representing 10-fold K_i_ showed fluorescent spot densities similar to the SNAP-D_2L_ receptor labeled with Alexa546-BG, indicating >90% labeling of the receptors by **1c** (Fig. 7, Supplementary Table S2, Supplementary Movie S4). The results of intensity distributions of monomers:dimers (74% : 26%, SNAP-D_2L_ labeled with **1c** and 71% : 29%, D_2L_ labeled with **1c**) suggest that the extent of dimerization was not influenced by the SNAP-tag (Fig. 7c,f).

To verify specific labeling of the D_2L_ receptors with the ligands **1c** and **2c**, CHO cells stably expressing SNAP-D_2L_ receptors were pretreated with 10 μM spiperone. Only a small number of stationary spots were found, adhered to the glass slide surface (nonspecific binding), but cell labeling was completely blocked and no moving fluorescent spots were observed at the plasma membrane (Fig. 7e).

An intensity distribution analysis to calculate the monomer/dimer ratio of D_2L_ receptors tagged with the bivalent fluorescent ligand **2c** was not possible, since this approach requires labeling of the receptor with a second fluorophore. Nevertheless, the low average diffusion coefficient 0.074 ± 0.016 μm^2^ s^−1^ (mean ± s.d., 6 cells) of D_2L_ receptors labeled with the bivalent ligand **2c** provided evidence for increased receptor dimerization (Supplementary Table S2).

#### Agonists and Antagonists

**Figure 8:**
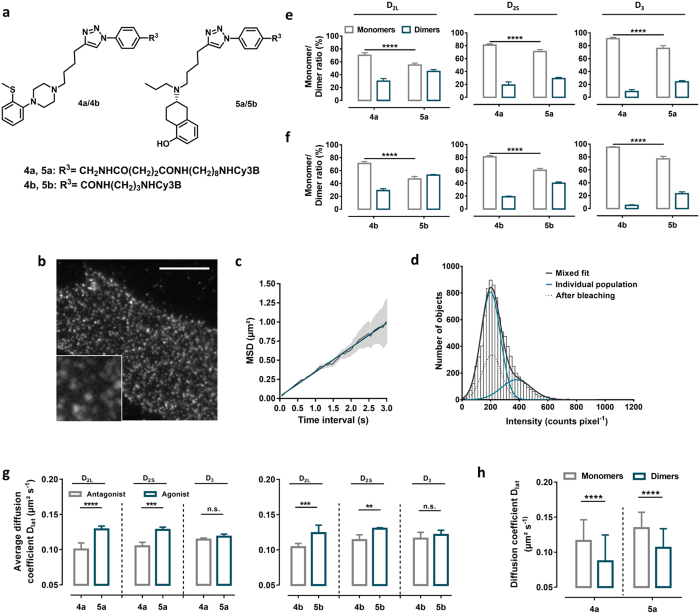
Influence of antagonists (4a,b) and agonists (5a,b) on D_2L_, D_2S_ and D_3_ receptor dimerization. (**a**) Chemical structures of monovalent fluorescent antagonists (**4a,b**) and agonists (**5a,b**). (**b**) Representative images a single CHO cell stably expressing the D_2L_ receptor, labeled with 4a (38 nM), visualized by TIRF-M. Scale bar, 10 μm. Insert correspond to a higher magnification image of the area in the white box. (**c**) Plot of average mean square displacement (MSD ± s.d.) versus the time interval (δt) of the receptor ligand complexes that were tracked (r^2^ = 0.99 – linear fit (blue)). (**d**) Intensity distribution of fluorescent spots identified over the first 10-frame time window of ligand receptor complex D_2L_-**4a**. (**e,f**) Monomer/dimer ratios of D_2L_, D_2S_ and D_3_ receptors labeled with the fluorescent antagonists **4a,b** and agonists **5a,b**, calculated from fitted fluorescence intensity distributions of fluorescent ligand receptor complexes using a mixed Gaussian model (compare to (**c**) and Supplementary Table S5). (**g**) Average diffusion coefficients (D_lat_) of the receptor complexes of the same cells analyzed in (**e**,**f**). Data in (**e**–**g**) represent mean ± s.d. *n* analysed cells (D_2L_: *n* = 18 for **4a**, 19 for **5a**, 14 for **4b** and 17 for **5b**, D_2S_: 13 for **4a**, 12 for **5a**, 19 for **4b** and 12 for **5b**, D_3_: 12 for **4a**, 13 for **5a**, 15 for **4b** and 10 for **5b**). (**e**) Representative effects of the size of D_2L_ receptor-ligand complexes on their rates of lateral diffusion. The diffusion coefficient of D_2L_-4a monomers (0.116 ± 0.030 μm^2^ s^−1^, *n* = 838 from 3 cells) is greater than that of the dimers (0.087 ± 0.037 μm^2^ s^−1^, *n* = 185 from 3 cells). This is also found for D_2L_-**5a** monomers 0.134 ± 0.023 μm^2^ s^−1^ (*n* = 1396 from 3 cells) and dimers 0.106 ± 0.027 μm^2^ s^−1^ (*n* = 558 from 3 cells). Data represent means ± s.d. Statistical analysis was performed by an unpaired *t*-test (**p-value < 0.01, ***p-value < 0.001, ****p-value < 0.0001, n.s. - not significant).

We chose fluorescent ligands to investigate the effect of agonists on dopamine receptor dimerization compared to antagonist-bound receptors. Accordingly, we designed and synthesized the fluorescent dopamine D_2 _receptor antagonists **4a,b** bearing a 1,4-disubstituted phenylpiperazine head group (1,4-DAP), a lipophilic, heterocyclic appendage for enhancing ligand affinity, an ω-amino acid-based linker of different lengths (6 and 16 atoms) and the fluorophore Cy3B (Fig. 8a, Supplementary Figs S13 and S14). The fluorescent agonists **5a,b** shared the same architecture (Fig. 8a) except the 1,4-DAP pharmacophore was replaced by that of the strong D_2_ agonist (S)-5-OH-DPAT (Fig. 8a, Supplementary Figs S15 and S16). **5a** and **5b** were potent agonists at the D_2L_ receptor (Supplementary Fig. S9b). The fluorescent ligands of type **4** and **5** were assessed for dopamine receptor affinity using radioligand binding studies, exhibiting low nanomolar to subnanomolar K_i_ values at the dopamine D_2L_ receptor (Supplementary Table S1). These four fluorescent ligands (**4a,b** and **5a,b**) were characterized on glass slides for their single molecule behaviour and were visible by TIRF-illumination as discrete fluorescent spots. The observed time traces of one-step photobleaching confirmed that single fluorescent m were imaged. The fluorescent intensity distribution of individual spots of these particular fluorescent ligands showed a predominant peak with an average intensity of approximately 200 counts pixel^−1^ (199 ± 78 counts pixel^−1^ **4a**, 190 ± 81 counts pixel^−1^ **4b**, 200 ± 79 counts pixel^−1^ **5a**, and 197 ± 80 counts pixel^−1^ **5b** (mean ± s.d.)) (compare with Supplementary Fig. S7a,b). CHO cells stably expressing the D_2L_ receptor were incubated with a 10-fold concentration of the K_i_ value of the corresponding fluorescent ligand **4a,b** and **5a,b** and were imaged by TIRF-M with equivalent photobleaching behaviour (Supplementary Table S4). To confirm specific labelling of these receptors by the fluorescent ligands **4a,b** and **5a,b**, CHO cells stably expressing D_2L_ were pretreated with 10 μM spiperone, when no cell-specific labeling and a small number of non-specifically adhered, immobile fluorophores could be identified (Supplementary Fig. S9a).

D_2L_ receptors labeled with the fluorescent antagonist **4a** could be identified as individual fluorescent spots moving rapidly on the plasma membrane (Fig. 8b). The intensity distribution of these fluorescent spots showed that the D_2L_ receptor ligand complex (D_2L_-**4a**) had a similar pattern to that with the fluorescent antagonist **1c**, and contained mainly two populations with the same ratio of monomers (70%) and dimers (30%) (Fig. 8c–f, Supplementary Table S5). Moreover, the average receptor diffusion coefficient, D_lat_ 0.100 ± 0.009 μm^2^ s^−1^ (mean ± s.d., 18 cells) was similar to that the D_2L_ receptor labeled with the fluorescent antagonist **1c** (D_lat_, 0.109 ± 0.006 μm^2^ s^−1^, mean ± s.d., 6 cells).

The dimer population of the D_2L_ receptors labeled with the fluorescent agonist **5a** was increased to 45% from the value of ca. 30% in the presence of antagonist **4a**. An identical behavior was observed for the fluorescent ligands with the shorter linkage between the pharmacophore and the fluorophore moieties (42% and 29% for **5b** and **4b**, respectively) (Fig. 8e,f, Supplementary Table S5).

To investigate if the extent of dopamine receptor dimerization is subtype specific, we extended our strategy to the structurally related human D_2S_ and D_3_ receptors. When viewed by TIRF-M, CHO cells stably expressing the D_2S_ or D_3_ receptor (receptor expression level (B_max_) of 970 ± 60 fmol mg^−1^ protein, for D_2_ _S_ and 1060 ± 60 fmol mg^−1^ protein, for D_3_, (mean ± s.e.m.) labeled with the corresponding ligands **4a,b** and **5a,b** (10-fold K_i_ value labeling concentration) the labeling pattern appeared similar to CHO cells expressing D_2L_ receptors.

Based on the distribution of individual fluorescent spot intensities of the fluorescent antagonists **4a,b** bound to D_2S_, and D_3_ receptors, we observed that 18% and 19% of labeled D_2S_ and 10% and 9% of the labeled D_3_ receptors form dimers, respectively (Fig. 8e,f, Supplementary Table S5). Agonist labeling with **5a** and **5b** increased the proportion of dimers and increased the average diffusion rate of all three dopamine D_2L_, D_2S_ and D_3_ receptor subtypes (Fig. 8g, Supplementary Table S5).

The agonist-induced increase in receptor mobility of D_2L_-**5a** monomers and dimers is unexpected but might be generated by changes in the conformation of the receptor that influence the mobility or its local environment (Fig. 8h). To confirm this result we incubated Alexa546-labeled SNAP-D_2L_ receptors with the reference D_2_ agonist S-(–)-5-OH-DPAT and the antagonist haloperidol. An agonist-induced increase of receptor dimerization and mobility was again observed, whereas the antagonist showed a monomer/dimer ratio that was identical to that of unliganded receptors (Supplementary Fig. S10).

## Discussion

We could visualize SNAP-tagged D_2L_ receptors in stably transfected CHO cells under TIRF illumination as individual, freely diffusing fluorescent spots that were evenly distributed at the cell surface. Transient formation of dimers with a lifetime of approximately 0.5 sec and a monomer-dimer ratio of about 70% to 30% was demonstrated, providing evidence that the D_2L_ receptor exists in a dynamic equilibrium between monomers and dimers. These results are consistent with single-molecule studies on other class A GPCRs^9^,^11^.

Recent studies have discussed whether the extent and the dynamics of dimerization can be modulated by the absence or presence and the nature of specific ligands^7^,^8^,^21^,^39^,^40^. Our data show that the extent of D_2L_ receptor dimerization could be significantly modulated in the presence of dopamine receptor ligands. Treatment with fluorescent agonists, but not antagonists, led to a significant increase of dimers, indicating ligand-specific promotion of attractive protein-protein interactions that determine the equilibrium. The receptor subtypes D_2S_ and D_3_ showed an analogous behavior upon antagonist and agonist binding, despite the extent of dimerization being subtype sensitive (D_2L_ > D_2S_ > D_3_ at an expression level of ca 1 pmol mg^−1^ protein, Supplementary Table S5).

Bivalent ligands that bridge two adjacent protomers display a promising strategy to specifically generate and target GPCR dimers, but their effect on the dynamics and the degree of dimerization is less well established^24^,^25^. Our results indicate that bivalent antagonists of type **2** significantly shift the equilibrium towards the dimeric state of D_2_ _L_ with 66% and 58% ligand-dimer complexes being observed in presence of **2a** and **2b**, respectively. The higher extent of D_2L_ receptor dimerization (ca. 70% of the receptor molecules being present as dimers) was also reflected in a decreased averaged diffusion coefficient of the receptor ligand complexes which is consistent with the increased radius of a dimer compared to the monomer. This indicates a ligand-induced physical interaction within the D_2L_ receptor dimer, with the decreased average diffusion coefficient of untagged D_2L_ receptors in presence of the fluorescent bivalent ligand providing additional evidence for an increase in dimerization. The effect depends on the presence of two D_2_-specific pharmacophores incorporated in the bivalent ligand, because an increase of dimerization was not observed upon the monovalent analogs **1a,b** and the control ligands **3a,b** composed of a pharmacophore with a complete spacer arm and a structurally disordered pharmacophore. Lifetime analysis of the receptor dimers provided evidence that bivalent ligands substantially modulate the lifetime of the investigated D_2L_ receptor dimers. Whereas dimerization has been shown to be a transient phenomenon with a mean lifetime of a dimer of approximately 0.5 s for unliganded receptors and receptors bound by monovalent antagonists, receptors bound to bivalent ligands were stable for longer than the complete tracking time (4 s for the longest tracks. The lifetime of the dimer might be controlled by the dissociation of the ligand, which is in the range of minutes, and not by the dynamics of the protein in the membrane.

Given that homomeric and heteromeric receptor complexes may exhibit unique functional characteristics compared to their constituent receptors and may have a more restricted distribution^41^, the ability of ligands to selectively target homomeric dimers in favor of monomeric and heteromeric complexes could have significant therapeutic implications.

The diameter of class A GPCR is approximately 6 nm, around 30-fold lower than the diffraction limit of light^42^. In recent years, the development of super-resolution methods allowed to overcome the diffraction limit and substantially enhanced the optical resolution down to the nanometer scale^43^,^44^,^45^. To further distinguish between random colocalization of two closely lying receptors and a physical interaction of two protomers in a receptor dimer, we applied cryogenic localization microscopy, a super resolution imaging technique by single-molecule localization at low temperature (4.3 K). After inducing membrane protrusion formation through exposure of the cells to hypo-osmotic stress conditions, SNAP-D_2L_ receptors in intact CHO cells were frozen, using cryo-immobilization which preserved the structures in a near native state and their behavior was compared to monomeric CD86 membrane proteins. The decreased photo-bleaching at lower temperature and the resultant increase of the localization precision is a beneficial side effect of this technique^28^,^29^,^46^.

Cryogenic localization microscopy allowed us to display SNAP-D_2L_ receptor dimers with high resolution and to measure the distance between their centers of mass. The determined distance of about 9 nm indicated a physical interaction of the protomers. The experimental distance is in good agreement with that predicted by a homology model of the dopamine D_2_ receptor homodimer which we developed based on the crystal structure of the β_1_ adrenergic receptor^47^.

## Methods

### Organic synthesis and characterization of ligands

Detailed schemes and conditions for the synthesis of the ligands **1b,c**, **2b,c**, **4a,b** and **5a,b** are provided in Supplementary Figs S11 to S16. Detailed methods and characterization for all compounds and precursors are provided in Supplementary Note S4.

### Plasmid constructions

A plasmid coding for the *N*-terminally SNAP-tagged human dopamine D_2L_ receptor (SNAP-D_2L_) was generated by replacing the β_2_AR coding region of a pSNAP_f_-β_2_ adrenergic receptor (SNAP-β_2_AR) (New England Biolabs). The human D_2L_ receptor was PCR amplified using primers designed to add SbfI and XhoI sites to the fragment termini. This was then ligated into the multiple cloning site downstream of SNAP-coding sequence in the plasmid pSNAP_f_. The absence of unwanted mutations was confirmed by sequencing. The construct was functional, as shown by radioligand binding and cAMP concentration-response curves. SNAP-CD86 and SNAP-CD28 were generated by exchange of D_2L_ of the SNAP-D_2L_ plasmid against CD86 and CD28, respectively. Plasmids coding for YFP-CD86 (human CD86 - truncated at R277) and CD28-YFP (YFP - F46L_L68V, human CD28 - truncated at R185) were kindly provided by Martin Lohse (Institute of Pharmacology and Toxicology and Bio-Imaging Center, University of Würzburg, Würzburg, Germany). CD86 or CD28 were PCR amplified and ligated into the plasmid pSNAP_f_ described above.

### Generation of stable CHO-K1 cell lines

Chinese hamster ovary cells (CHO-K1) were cultured in DMEM/F-12, supplemented with 10% FCS, 1% penicillin-streptomycin, 2 mM L-glutamine at 37 °C and in the presence of 5% CO_2_. For the generation of the stable cell line expressing SNAP-D_2_ _L_, SNAP-CD86 or SNAP-CD28, CHO-K1 cells seeded in 6-well dishes and grown to 50% confluence were transfected with Mirus TransIT^®^-2020 (MoBiTec), following the manufacturer’s protocol. After 48 h, the medium was changed to medium supplemented with 1200 μg ml^−1^ geneticin G418 (Gibco) to initiate selection of antibiotic-resistant cells. Several resistant clones were isolated by limiting dilution, and characterized for the SNAP-D_2L_ receptor by radioligand saturation experiments. The protein expression of the CD86 and CD28 was validated by TIRF microscopy. For the maintenance of stably transfected cell lines, the concentration of G148 was reduced to 800 μg ml^−1^ geniticin G418 to prevent the reversion of transfected CHO-K1 cells to a non-transfected state.

### Cell culture

Chinese hamster ovary cells (CHO-K1) stably expressing the human dopamine D_2L_^48^, D_2S_^48^ receptor, the SNAP-D_2L_ receptor or the SNAP-CD86 and SNAP-28 proteins were maintained in DMEM/F12 medium supplemented with 10% FCS, 2 mM L-glutamine, 1%, penicillin-streptomycin, and 800 μg mL^−1^ geneticin. Dihydrofolate reductase gene-deficient CHO-K1 cells stably expressing human D_3_ receptors (D_3_)^49^ were grown in DMEM medium containing 4.5 g L^−1^ glucose, supplemented with 10% heat-inactivated dialyzed FBS, MEM amino acid supplement, 2 mM L-glutamine and 1%, penicillin-streptomycin. All cells were grown at 37 °C and in 5% CO_2_.

### Membrane preparation

Membrane preparations were obtained using the methods described previously^50^. In brief, CHO cells stably expressing the D_2L_, D_2S_, D_3_ or SNAP-D_2L_ were washed with 10 mL ice cold phosphate-buffered saline (PBS, pH 7.4), treated with harvest buffer (10 mM Tris-HCl, 0.5 mM EDTA, 5.4 mM KCl, 140 mM NaCl, pH 7.4), and dissociated using a cell scraper. Subsequently they were pelleted at 200 g for 8 min at 4 °C and resuspended in 10 mL of ice cold homogenate buffer (50 mM Tris-HCl, 5 mM EDTA, 1.5 mM CaCl_2_, 5 mM MgCl_2_, 5 mM KCl, 120 mM NaCl, pH 7.4). Cell suspensions were then lysed using an Ultraturrax (20000 rpm, 5 times for 5 s) and centrifuged at 50000 g for 15 min. After resuspending the membranes in the binding buffer (50 mM Tris-HCl, 1 mM EDTA, 5 mM MgCl_2_, 100 μg mL^−1^ bacitracin, 5 μg mL^−1^ soybean trypsin inhibitor, pH 7.4) they were homogenized with a glass−Teflon homogenizer for 7 min at 4 °C. The homogenized membranes were shock-frozen in liquid nitrogen and stored at −80 °C until usage. The protein concentration was determined by the Lowry method^51^ using bovine serum albumin as a standard.

### Receptor binding studies

Receptor binding studies were carried out as described previously^27^,^50^,^52^. The radioligand [^3^H]spiperone (specific activity 80.6 Ci mmol^−1^) (Perkin Elmer) was used in saturation experiments to determine the K_d_ and B_max_ values for the membrane preparations of stably transfected CHO cells expressing the human D_2L_, D_2S_^48^, D_3_^49^ and SNAP-D_2L_ receptors, respectively,. The K_i_ values for the compounds were obtained by competition experiments. In brief, the assay were carried out in the 96-well plates at protein concentration of 1–8 μg assay^−1^ tube in a final volume of 200 μL and [^3^H]spiperone at final concentrations of 0.125–0.200 nM for D_2L_, D_2S_, D_3_ and SNAP‐D_2L_ receptors. The K_D_ values of [^3^H]spiperone for D_2L_, D_2S_, D_3_ and SNAP-D_2L_ receptors, were 0.053–0.085, 0.067–0.120, 0.095–0.180 and 0.110–0.130 nM respectively, and the corresponding B_max_ values were in the range of 610–640, 1500–4800, 4200–6450 and 2000–2100 fmol mg^−1^.

### Data analysis for receptor binding studies

Analysis of the saturation experiments were performed using a nonlinear regression analysis of the data for the determination of K_D_ and B_max_ values using PRISM (GraphPad Software). Competition curves were fitted to a sigmoid curve by nonlinear regression analysis in which the log IC_50_ value and the Hill coefficient were free parameters. IC_50_ values were transformed to K_i_ values according to the equation of Cheng and Prusoff 53.

### Adenylyl cyclase inhibition assay

Bioluminescence based cAMP-Glo™ assays (Promega) were performed according to the manufacturer’s instructions. Briefly, CHO cells stably expressing the D_2L_ or SNAP-D_2L_ receptor were seeded into a white half-area 96-well plate (5000 cells well^−1^) 24 h prior to the assay. On the days of the assays cells were washed with phosphate buffered saline (PBS, pH 7.4) to remove traces of serum and incubated with various concentrations of compounds in the presence of 20 μM forskolin in serum-free medium that contained 500 μM IBMX and 100 μM Ro 20–1724, pH 7.4. After 15 min of incubation at 25 °C cells were lysed with cAMP-Glo lysis buffer, the kinase reaction was performed with a reaction buffer containing PKA and finally an equal volume of Kinase-Glo reagent was added. Bioluminescence was read on a microplate reader Victor^3^V (Perkin-Elmer). The experiments were performed at least three times per compound.

### D_2L_ receptor activation

Ligand-induced activation of the human D_2L_ receptors was studied employing inositol phosphate (IP) accumulation assays as described^54^,^55^. Briefly, HEK 293 cells were transiently co-transfected with cDNA encoding for D_2L_ and the hybrid G protein Gα_qi5_ (Gα_q_ protein with the last five amino acids at the C terminus replaced by the corresponding sequence of Gαi; gift from the J. David Gladstone Institutes). Twenty-four hours after transfection, cells were transferred into 24-well plates. After adding *myo*-[^3^H]inositol (specific activity = 22.5 Ci mmol^−1^, PerkinElmer) and incubation for 15 h, medium was aspirated, the cells were washed with serum-free medium supplemented with 10 mM LiCl, and test compounds 37 °C. Then, cells were lysed by adding 0.1 M NaOH. After neutralization with formic acid, the cell extract was separated by anion-exchange chromatography using an AG1-X8 resin (Bio-Rad) by washing and finally eluting total IP directly into scintillation counting vials. Radioactivity was determined by scintillation counting in a Beckman LS 6500 (Beckman). Data were analyzed by normalizing disintegrations per minute (d.p.m.) values; this was done by setting the data for non-stimulated receptor (buffer) equal to zero and the effect for quinpirole equal to 100%.

### Glass slide cleaning

18 mm no. 1 glass slides (Assistent) were extensively cleaned to remove any background fluorescence. First, they were sonicated in a solution containing 12% Decon 90 for 1 h. After three washes with Milli-Q filtered water, they were further sonicated in a solution of 5 M NaOH for 1 h and were washed again three times with Milli-Q filtered water. Glass slides were then dried followed by sonication in chloroform for 1 h. Cleaned glass slides were dried and stored in 100% ethanol until use.

### Labeling and preparation for single-molecule TIRF-M imaging

24 h before the TIRF-experiment dried and cleaned glass slides were placed in a 12 well plate and were coated by incubation with 20 μg ml^−1^ fibronectin (Sigma–Aldrich) in sterile PBS for 1 h at 37 °C. After coating, fibronectin was aspirated and the glass surface was rinsed one time with sterile PBS. CHO cells expressing SNAP-D_2L_, SNAP-CD86, SNAP-CD28, D_2L_, D_2S_ or D_3_ receptors were seeded on coated glass slides in phenol-red-free DMEM/F12 supplemented with 10% FCS and were allowed to adhere overnight at 37 °C and 5% CO_2_.

#### SNAP-tag labeling

Cells were washed two times with phenol red-free DMEM/F12 supplemented with 10% FBS and were labeled 30 min at 37 °C with 1 μM Alexa546-BG (SNAP-Surface^®^ Alexa Fluor^®^ 546; New England Biolabs). Subsequently, cells were washed three times with phenol red-free DMEM/F12 supplemented with 10% FBS, each time followed by 5 min incubation at 37 °C.

For the ligand treatment experiments, the indicated concentration of the corresponding ligand in phenol red-free DMEM/F12 supplemented with 10% FBS were added to stably transfected CHO cells, which were ready for imaging, for 1 h before imaging.

#### Fluorescent-ligand labeling

Cells were washed two times with phenol red-free DMEM/F12 supplemented with 10% FBS, labeled with a 10-fold K_i_ value concentration of the corresponding fluorescent ligand (Supplementary Table S1) and incubated (**1c**, **2c** and **4a,b** at 37 °C, 5% CO_2_ and **5a,b** at room temperature) for 1 h. Specific labeling of the fluorescent ligands was confirmed by pre-incubation with 10 μM spiperone (a potent non-fluorescent dopamine D_2_ receptor antagonist) for 2 h, followed by incubation with fluorescent ligand described above.

Subsequently after labeling, cells were washed three times with phenol red-free DMEM/F12 supplemented with 10% FBS. Glass slides with labeled cells were placed in a custom-built imaging chamber (volume = 500 μL). washed two times with imaging buffer (137 mM NaCl, 5.4 mM KCl, 2.0 mM CaCl_2_, 1.0 mM MgCl_2_, and 10 mM Hepes, pH 7.4). Finally the imaging chamber was refilled with fresh imaging buffer and mounted immediately on a microscope stage for TIRF-M imaging.

### TIRF-M imaging system

Experiments were performed at room temperature (24.0 ± 0.3 °C) on a motorized Nikon TI-Eclipse inverted microscope equipped with a 100x, 1.49 NA oil-immersion objective. Fluorescent dyes were excited using a Nikon D-Eclipse C1 laser box with 561 nm laser for TIRF microscopy. Excitation filter at 561/14 nm employing dichroic long-pass mirror (cut-off wavelength 561 nm) was used. The emitted light was passed through emission bandpass filter 609/54 nm (Semrock Rochester), and was projected onto a water-cooled (Polar Series Accel 250 LC, Thermo Scientific) EM-CCD camera (512 × 512 FT, DU-897, Andor) to −98 °C. The microscope control and image acquisition were performed by the NIS Elements software (Nikon Instruments). To ensure homogenous illumination, only the central quarter of the chip (300 × 300 pixel) was used for imaging analysis. The gain of the EM-CCD camera was set and kept constant at 300, binning at 1 × 1, BitDepth at 14 bits, readout speed at 10 MHz and image sequences (300–500 frames, except 1000 frames for photobleaching experiments) were acquired with an exposure time of 50 ms, resulting in the frame rate of 19.32 fps (frames per second). Under these conditions a representative photobleaching half-life time t_1/2_ of 6.23 ± 0.43 (mean ± s.d.) was determined for Alexa564 labeled SNAP-D_2L_ receptors (Supplementary Fig S3c) The evanescent penetration depth was calculated as ~80 nm.

### Data analysis for the TIRF-M imaging

The image analysis procedures have been described in detail previously^9^,^56^,^57^. An automated single particle tracking (ASPT) algorithm^58^ implemented in custom-written software, GMimPro (www.mashanov.uk), was used to identify and track individual fluorescent spots on sequential video frames. ASPT settings used were FWHM300nm, R5, L20, Q10 and C999. Output from this image analysis gave fluorophore centroid positions (with 25 nm precision), integrated fluorophore intensities, total fluorophore number and trajectory lifetime. Spatial trajectories and intensity trajectories (i.e. intensities vs. time) were then further analysed to determine: the lateral diffusion coefficient, *D*_lat_, which was derived from averaged mean squared displacement (MSD) versus time interval (δt), the average fluorescent spot density (spot μm^−^^2^) and distribution across the cell membrane and the intensity distribution of individual fluorescent spots and intensity fluctuation with time as well as the lifetime of colocalization. The *D*_lat_ was measured for each cell separately and the average value (average *D*_lat_, mean ± standard deviation (s.d.)) was calculated for an indicated number of cells. Slow-moving objects D_lat_ < 0.02 μm^2^ s^−1^ were excluded from the analysis and only cells with a mobile trajectory fraction (trajectories with a D_lat _> 0.02 μm^2^ s^−1^) over 0.75 were used for further analysis. Intensity distributions of fluorescent spots identified over the first 10-frame time window of TIRF illumination were fitted with a sum of Gaussian distributions (with standard deviation proportional to square root of intensity level) using the PRISM routine. The ratio of the areas under the Gaussian curves was calculated to give the percentage of the underlying component^57^. Unpaired two-tailed Student´s t-tests were used for statistical significance.

### Determination of the SNAP-tag labeling efficiency

CHO cells stably expressing the SNAP-D_2L_ receptor were seeded on fibronectin (20 μg ml^−1^) pre-coated 3.5 cm CELLview™ cell culture dishes with an integrated glass bottom (Greiner, surface treatment TC) with a density of 1 × 10^5 ^cells and incubated overnight in phenol-red-free DMEM/F12 supplemented with 10% FBS.

Medium was removed and the cells were labeled with increasing concentrations of Alexa564-BG (0–3 μM) as described above. An adequate amount of randomly chosen cells (*n* = 15–30) per concentration was imaged by TIRF-microscopy. One image sequences of 5 frames cell^−1^ was acquired with an exposure time of 50 ms. Further imaging settings were kept identical as described above. Brightfield images were captured and used as a guide for non- or weakly-labeled cells.

### Kinetic fluorescent ligand binding studies

For live-cell kinetic experiments to characterize the association and dissociation kinetics of fluorescent ligands, CHO cells stably expressing the SNAP-D_2L_ receptor were prepared as described above for the determination of the SNAP-tag labeling efficiency.

#### Determination of the association rate

Medium was replaced by fresh DMEM/F12 supplemented with 10% FBS and the fluorescent ligand was added at a concentration representing a 10-fold K_i_ value. The whole cell fluorescence at 37 °C was measured at times up to 210 min.

#### Determination of the dissociation rate

Medium was replaced by fresh DMEM/F12 supplemented with 10% FBS and the fluorescent ligand was added at a concentration representing 10-fold K_i_ value and incubation was continued for 60 min at 37 °C to reach equilibrium binding. Subsequently, the cell culture dishes were placed on the microscope stage and the incubation medium was replaced by spiperone with a final concentration of 5 μM in imaging buffer. The moment of spiperone addition was considered at t = 0 min and bound fluorescent ligand to a whole cell was imaged at multiple time intervals post t = 0 min (5 min to 340 min). An adequate amount of cells was imaged at each time interval by TIRF-microscopy.

### Calculation of the background corrected mean fluorescence intensity of single cells and analysis

Calculation of the mean background corrected fluorescence intensity *I(t)* (arbitrary units) at time *t* of single cells for live cell kinetic, SNAP-tag labeling efficiency and photobleaching experiments were performed as follows. Regions of interest (ROI) were drawn around the membrane of an individual fluorescent cell (ROI_cell_) and the background (ROI_background_) outside the cell using Fiji software^57^ (Supplementary Fig. S1b). The total mean intensity over the entire cell area (*I(t)*_*total cell*_) and the mean intensity over the background area (*I(t)*_*bg*_) were measured and *I(t)* were calculated as *I(t)* = *I* (*t*)_*total cell*_ − *I* (*t*)_*bg*_. Data were imported into PRISM and were fitted as stated for each condition by a one-phase association curve for ≥11 cells from three independent experiments per condition. The output provided the rate constant (*k*), the time constant τ (mean fluorescent lifetime) and the half-life time (t_1/2_) defined as k^−1^ and ln2 k^−1^, respectively.

### Labeling and preparation for cryogenic localization microscopy

Fused silica cover slips (7.0 × 7.0 × 0.2 mm, UV grade, Siegert Wafer GmbH) were cleaned by sonication using detergents (deionized water and soap “Frosch”), and alternating rinsing with deionized water and sonication in non-halogenated solvents (acetone, ethanol, 2-propanol, in that order) followed by sonication in Piranha solution (1:1 SO_4_ and 30% H_2_O_2_). Afterwards, the cover slips were stored in deionized water before a treatment in 5% hydrofluoric acid (HF) for 5 min in order to increase adherence of the cells to the cover slips. The cover slips were then dried with nitrogen (5N) and again rinsed with deionized water and ethanol before usage.

The clean and dry HF-treated fused silica slides were then coated with fibronectin like the coating procedure described above (Labeling and preparation for single-molecule TIRF-M imaging). CHO cells stably expressing SNAP-D_2L_ and SNAP-CD86 receptors, respectively, were seeded on coated glass slides in phenol-red-free DMEM/F12 supplemented with 10% FCS and were allowed to adhere overnight at 37 °C and 5% CO_2_. Hypoosmotic stress conditions to induce membrane protrusion formation were attained by incubation in hypo-osmotic PBS (108 mOsm) for 2 h at 37 °C and 5% CO_2_. After incubation, the cells were labeled with Alexa546-BG like the SNAP-tag labeling procedure described above. To exclude artefacts from the HF treatment, the above described procedure, but with 20 min in 5% HF to account for slower etching of borosilicate glass compared to fused silica, was applied to 18 mm, no. 1 glass slides (Assistent) (TIRF-M slides) which were employed for TIRF-M control experiments with CHO cells, stably expressing the SNAP-D_2L_ receptor and labeled with Alexa546-BG.

### Cryogenic localization microscopy imaging setup

A schematic drawing of the experimental setup is shown in Supplementary Fig. S4. The experiments were performed on a homebuilt epi-fluorescence microscope. A Coherent Sapphire OPSL laser (λ = 532 nm) was coupled into a polarization maintaining fiber for beam clear up before it was out-coupled and collimated. A 532-10 band pass excitation filter was used for spectral clear up. The polarization was then adjusted using two wave plates, so that the light is circularly polarized before the microscope objective. A wide-field lens (f = 400 mm) focused the laser beam via a 4f telescope (f = 350 mm) into the backfocal plane of the microscope objective (Zeiss Neofluar 63x LWD, 0.75 NA). A glass wedge at low angle of incidence (about 5°) was used as a beam splitter. The sample is mounted in the vacuum chamber of a liquid helium flow cryostat (Janis ST-500) on a copper cold finger and cooled to liquid helium temperature (T = 4.3 K). The laser power was adjusted to about 5 mW before the microscope objective. The fluorescence is detected in epi-mode. A 538 long pass filter is used as detection filter before an f = 300 mm lens focuses the light on the EM-CCD camera (Andor iXon 897). Movies were recorded at a frame rate of 20 fps in frame transfer mode with EM gain = 2400.

### Data analysis for the cryogenic localization microscopy

The 3B analysis was performed using the ImageJ PlugIn provided by Rosten *et al*.^59^. Relatively large data sets (50 × 50 px up to 70 × 70 px, 1000 frames) were processed using default settings and measured values for the PSF FWHM. The analysis ran for >200 iterations to ensure convergence (runtime >7 days on an i7 3.4 GHz workstation).

We then calculated pairwise distances (Euclidian metric) from the identified positions. These histograms were corrected by subtracting a simulated histogram that was constructed in the following way: The same number of emitters was randomly placed on a three-dimensional cylinder of 150 nm diameter^60^ and the accumulated membrane protrusion length. Pairwise distances were then histogrammed after computing a two-dimensional projection.

### Dimer modeling

As a starting structure, our recently described homology model of the D_2_ receptor based on the D_3_ crystal structure was used^61^. The Swiss-PdbViewer^62^ was used to place a SNAP protomer (PDB 3KZY) in different positions on the *N*-terminal side of the D_2_ receptor model with its C-terminus allocated to the receptor. Missing residues, including unresolved SNAP residues, four additional linker residues and unresolved *N*-terminal D_2_ residues were modelled manually. The loop was created and refined by means of the Swiss-PdbViewer loop database. The dimer models were generated by superimposing two identical SNAP-D_2_ protomers with the crystal structure of the β_1_-AR dimer (PDB 4GPO)^63^. Subsequently, the resulting dimer models were submitted to energy minimization as described previously^61^. The figure was prepared using UCSF Chimera package 1.10^47^.

## Additional Information

**How to cite this article**: Tabor, A. *et al*. Visualization and ligand-induced modulation of dopamine receptor dimerization at the single molecule level. *Sci. Rep.* **6**, 33233; doi: 10.1038/srep33233 (2016).

## Supplementary Material

Supplementary Information

Supplementary Movie S1

Supplementary Movie S2

Supplementary Movie S3

Supplementary Movie S4
