# Supplementary material for: Visualization and ligand-induced modulation of dopamine receptor dimerization at the single molecule level
Source: Sci Rep. 2016 Sep 12;6:33233. doi: 10.1038/srep33233 (PMC5018964; doi:10.1038/srep33233)
Supplement: Supplementary Information [file srep33233-s1.pdf]

Visualization and ligand-induced modulation of dopamine receptor dimerization at the single molecule level

Alina Tabor [1], Siegfried Weisenburger [2,5], Ashutosh Banerjee [1], Nirupam Purkayastha [1], Jonas M. Kaindl [1], Harald Hübner [1], Luxi Wei [2], Teja W. Grömer [3], Johannes Kornhuber [3], Nuska Tschammer [1], Nigel J. M. Birdsall [4], Gregory I. Mashanov [4], Vahid Sandoghdar [2], and Peter Gmeiner [1]

[1] Department of Chemistry and Pharmacy, Emil Fischer Center, Friedrich-Alexander University, Schuhstraße 19, 91052 Erlangen, Germany

[2] Max Planck Institute for the Science of Light and Department of Physics, Friedrich-Alexander University, Günther-Scharowsky-Straße 1/ Bldg. 24, 91058 Erlangen, Germany

[3] Department of Psychiatry and Psychotherapy, Friedrich-Alexander University, Schwabachanlage 6, 91054 Erlangen, Germany

[4] The Francis Crick Institute, Mill Hill Laboratory, Mill Hill, London NW7 1AA, UK

[5] Current address: The Rockefeller University, New York, NY 10065, USA.

Supplementary Information (SI)

Supplementary Note S1: Single molecule validation of Alexa546-BG fluorophores.

Our TIRF-M imaging system was validated by observing single molecules of the cell-impermeable Alexa Fluor 546 BG (Alexa546-BG) adhered to a glass slide. Alexa546-BG appeared as well-dispersed diffraction-limited fluorescent spots, and the intensity profile had the expected spot size (full width at half maximum) of ~ 300 nm (Supplementary Fig. S2a,b). These fluorescent spots had a sufficiently high signal-to-noise ratio to be reliably identified and automatically detected. The observed one-step photobleaching confirmed that single molecules were imaged (Supplementary Fig. S2c,d and Supplementary Movie S2). The fluorescence intensity of individual spots (n = 2171) was normally distributed according to a single Gaussian function. Besides a largely predominant peak with an intensity of 198 ± 81 counts pixel^{-1} (mean ± s.d.), there is also a small peak of approximately twice that intensity, which is likely due to random colocalization of two fluorophores below the resolution limit (Supplementary Fig. S2e).

Supplementary Note 2: Molecular modeling.

Molecular modeling was used to estimate the distance between SNAP-tag fused dopamine D_2 receptors when associated as dimers. In Supplementary Fig. S7g, the distance between the Alexa546 fluorophores bound to the Cys145 residues in the two SNAP-tags was depicted. We were not able to predict one exact value due to the flexible loop linking the SNAP-tags and the dopamine D_2 receptors. However, the possibility to model this loop using different models with various distances between the SNAP-tags allowed the prediction of an approximate distance range. We created models with increasing distance between the SNAP-tags. The estimated range was defined by the model showing the smallest and the model with the highest distance. Using the modeling software Swiss-PdbViewer34 the loops were created. The distance between the sulfur atoms of the two Cys145 residues was measured. Positioning the two SNAP-tags at their closest proximity, while avoiding steric clashes, showed 2.8 nm between the two cysteine residues. The highest measured distance, in a model at which the Swiss-PdbViewer was able to form the loop was 12.7 nm. The preliminary investigation only considered one dimer orientation based on the β_1 adrenergic receptor[1] and only different symmetrical orientations of the SNAP-tags along one direction.

Supplementary Note S3: Single molecule validation of the fluorescent monovalent ligand 1c and bivalent ligand 2c.

Both fluorescent ligands were characterized on glass slides for their single molecule behavior. They were imaged by TIRF-M as discrete fluorescent spots and the observed time traces of one-step (**1c**) and two-step (**2c**) photobleaching confirmed that single fluorescent ligands were imaged (Supplementary Fig. S7a,c). The fluorescent intensity distribution of individual spots of both fluorescent ligands showed a predominant peak with an average intensity of 196 counts pixel^{-1} ± 78.3 (mean ± s.d.) for **1c** and approximately double intensity of 396 counts pixel^{-1} ± 110 (mean ± s.d.) for **2c** which is labeled with two Cy3B dye molecules (Supplementary Fig. S7b,d).

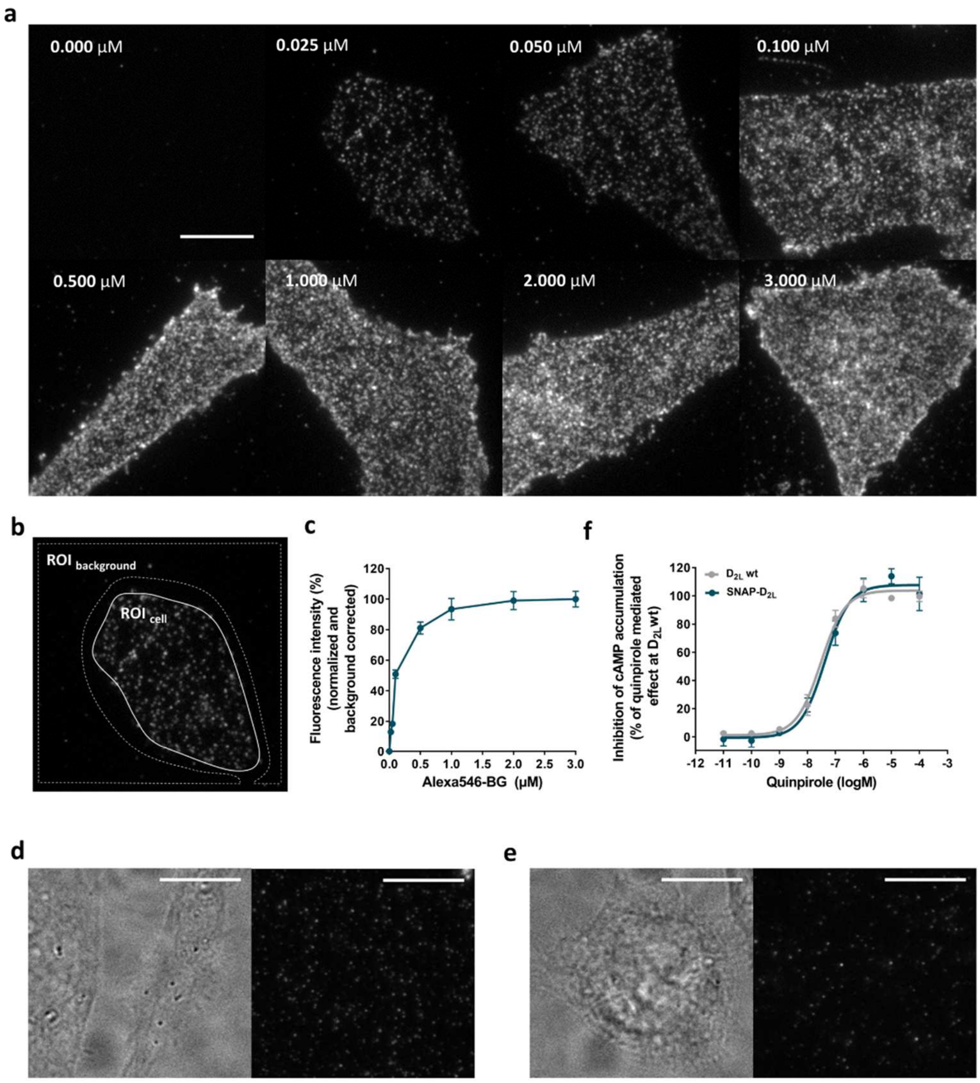

Supplementary Fig. S1 | Validation of the SNAP-tag labeling and functional characterization the SNAP-D_{2L} construct. (**a**) Representative TIRF-images of CHO cells stably expressing SNAP-D_{2L} and stained with different concentrations of Alexa546-BG. Scale bar, 10 µm. (**b**) Selection of regions of interest (ROIs) for the calculation of the background corrected mean fluorescence intensity of a single cell (see Methods section - Calculation of the background corrected mean fluorescence intensity of single cells and analysis). (**c**) Efficiency of the SNAP-tag labeling. CHO cells stably expressing SNAP-D_{2L} were stained with different concentrations of Alexa546-BG. Data points represent background corrected mean fluorescent intensities of 18 -34 cells (mean ± s.e.m) of each Alexa546-BG labeling concentration and normalization to the maximum fluorescence (100 %) of this data set from one representative experiment. (**d**, **e**) Validation of SNAP-tag specific labeling by Alexa546-BG. Representative brightfield (left) and TIRF-M image (right) of a CHO cell stably transfected with the wild type (wt) D_{2L} receptor lacking the SNAP-tag (**d**) and non-transfected CHO cells (**e**) incubated with Alexa546-BG (1µM). Scale bars, 10 µm. No cell-surface staining was present. (**f**) Functional characterization of the SNAP-tagged dopamine D_{2L} receptor measured by inhibition of cAMP accumulation. No substantial differences of the SNAP-D_{2L} receptor compared to the wild-type D_{2L} receptor were observed in potency and efficacy of quinpirole to inhibit cAMP accumulation in a concentration-dependent manner in response to ligand binding. cAMP assay was performed with intact living CHO cells, stably expressing the SNAP-D_{2L} or the wt D_{2L} receptor. The cAMP production was stimulated with 20 µM forskolin. Normalized curves from three experiments, each performed in triplicate, with error bars representing s.e.m. are shown. Intrinsic activity was determined relative to that of the reference substance quinpirole at the wt D_{2L} receptor.

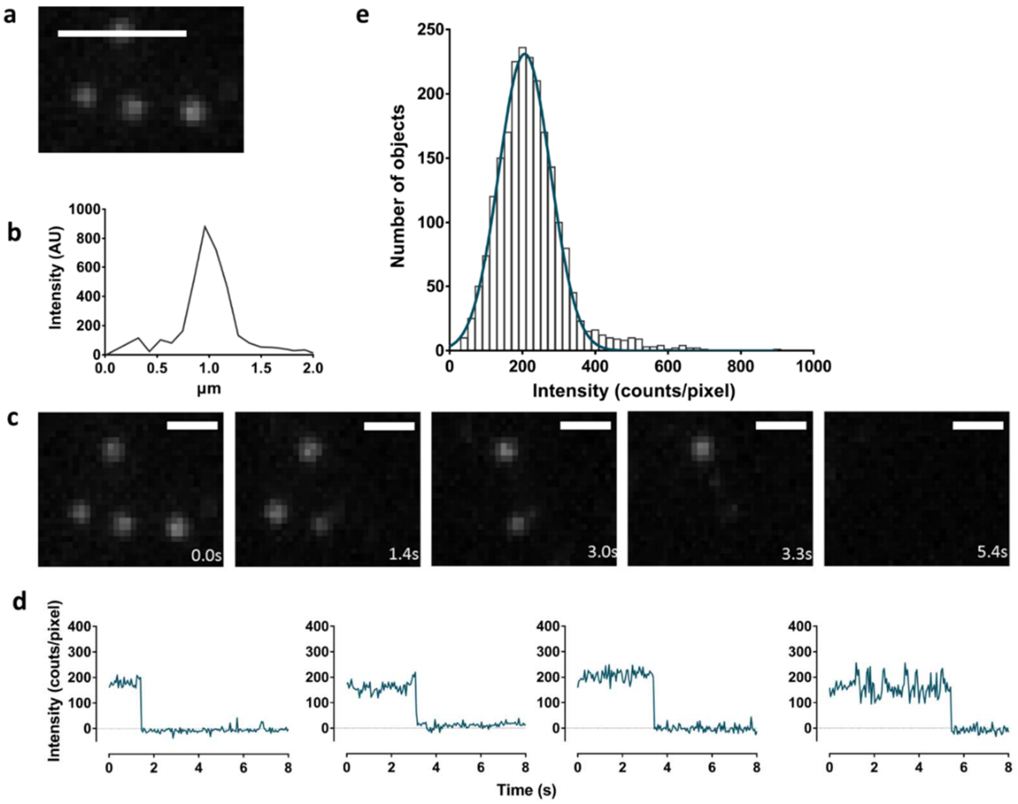

Supplementary Fig. S2 | Detection and characterization of single Alexa546-BG fluorophores by TIRF-M. (**a**) A single (50 ms exposure) TIRF image of diffraction-limited fluorescent spots of Alexa546-BG fluorophores adhered to a clean glass slide. (**b**) The intensity profile of an individual Alexa546-BG fluorophore marked by a white bar in **a**. (**c**) Single fluorescent spots of Alexa546-BG fluorophores showed photobleaching during laser exposure. Scale bar, 1 µm. (**d**) Four representative time courses of the Alexa546-BG fluorescent spots shown in **c**. (**e**) Distribution of the fluorescence intensity of single diffraction-limited Alexa546-BG fluorophores (n = 2171). Data were fitted with a single Gaussian model. Besides a largely predominant peak, an additional small peak of approximately double intensity was present, likely due to the random colocalization of two fluorophores.

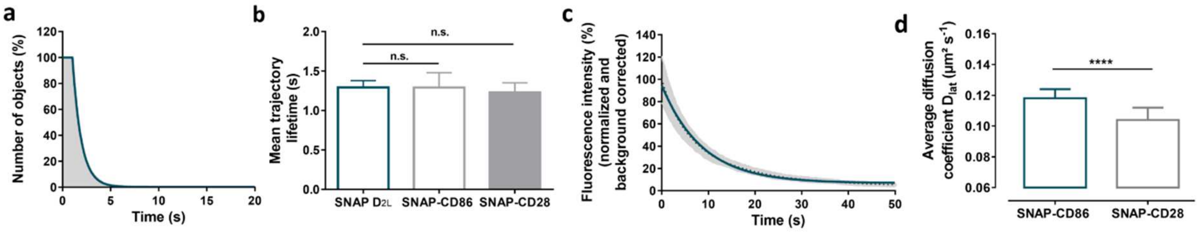

Supplementary Fig. S3 | Tracking and diffusion of Alexa546 labeled SNAP-D_{2L} receptors, monomeric SNAP-CD86 and dimeric SNAP-CD28 control proteins. (**a**) Plot of the trajectory lifetime of SNAP-D_{2L} receptors shown in Figure 1 d was fitted with a one-phase exponential decay function to determine the mean trajectory lifetime τ. (**b**) Mean trajectory lifetimes τ of the tracked SNAP-D_{2L} receptors (1.29 ± 0.09 s, 8 cells) compared to the monomeric and dimeric control proteins SNAP-CD86 (1.20 ± 0.19 s, 18 cells) and SNAP-CD28 (1.24 ± 0.12 s, 10 cells) which did not differ significantly. Data represent mean ± s.d.. Statistical analysis was performed by an unpaired *t*-test. n.s. not significant (*p*-value = 0.97 for SNAP-D_{2L} vs SNAP-CD86 and 0.25 for SNAP-D_{2L} vs SNAP-CD28). (**c**) Photobleaching of Alexa546 labeled SNAP-D_{2L} receptors under TIRF-illumination. Therefore the mean intensity over the entire cell area over time was measured; shown are the normalized mean fluorescent intensity of 10 cells (dotted line) ± s.d (shaded area) as well as a one-phase exponential fit (blue line) to determine the photobleaching rate of 0.14 ± 0.0008 s^{-1} (half-life time $t_{1/2}$ of 6.23 ± 0.43 s of 14 cells (mean ± s.d)). (**d**) Average diffusion coefficients (D_{lat}) of the dimeric SNAP-CD28 and monomeric SNAP-CD86 control proteins of 0.104 ± 0.008 µm² s^{-1} and 0.118 ± 0.006 µm² s^{-1} , respectively (mean ± s.d) (n = 6603, 9 cells – SNAP-CD86; n = 3999, 7 cells – CD28). The dimeric control protein SNAP-CD28 diffused more slowly compared to the monomeric control protein SNAP-CD86. The difference is statistically significant by an unpaired t-test (*****p*-value < 0.0001) and showed that the receptor mobility is negatively correlated with the size of the receptor complexes.

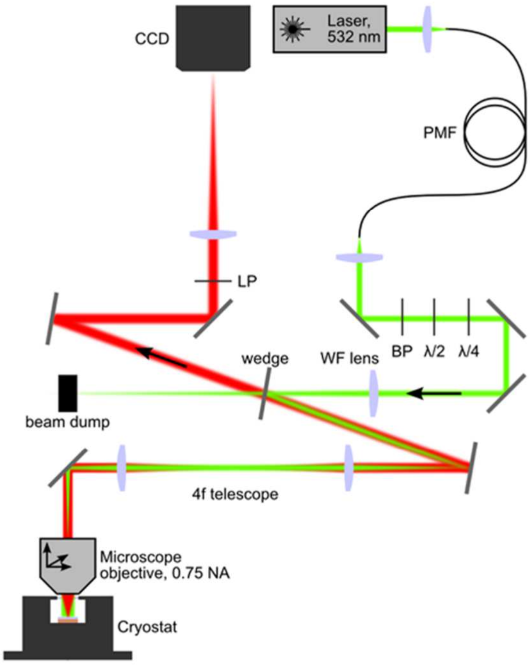

Supplementary Fig. S4 | Schematic drawing of the cryogenic and optical setup. PMF: Polarization maintaining fiber; BP: Band pass excitation filter; WF: wide-field; LP: Long pass detection filter. See Methods section for details.

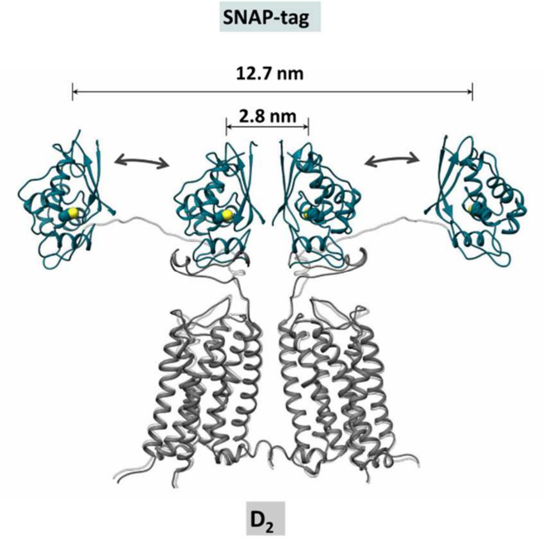

Supplementary Fig. S5 | Structural homology model of the SNAP-D_{2L} receptor dimer. Structural homology models of the dopamine D_2 receptor dimer fused to SNAP-tags (PDB 3KZY) in two different conformations of the linking loops suggesting minimal and maximal distances between the fluorophores. Model with SNAP-tags colored in blue and dopamine D_2 receptors colored in dark-grey (yellow spheres represent Cys145 residues in the SNAP-tags)**.** The homodimeric orientation was built on the basis of the β_1 adrenergic receptor crystal structure (PDB 4GPO)[2] to mimic an existing dimer structure. The Alexa546 fluorophores are positioned at the interacting residue Cys145 of the SNAP-tag [3].

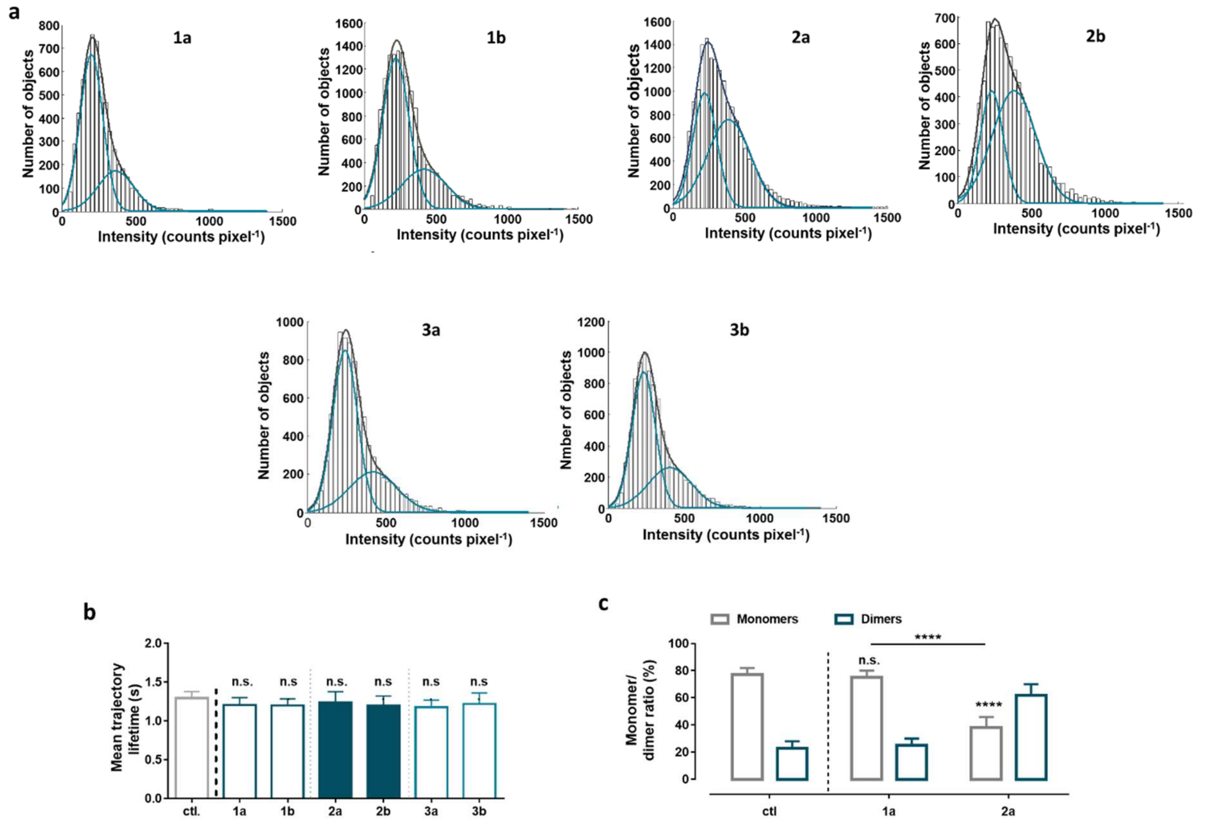

Supplementary Fig. S6 | Influence of monovalent (1a,b), bivalent (2a,b) and bivalent control (3a,b) dopamine D_2 receptor antagonists on receptor dimerization and mobility. (**a**) Representative intensity distributions of fluorescent spots identified over the first 10-frame time window of TIRF illumination of CHO cells, stably transfected with SNAP-D_{2L} receptor and labeled with a 10-fold K_i-value concentration of the corresponding ligand. Data were fitted with a mixed Gaussian model (sum of two Gaussian functions – mixed fit (grey), individual population (blue)). (**b**) Mean trajectory lifetimes of the tracked ligand-free SNAP-D_{2L} receptors (ctl.) and SNAP-D_{2L} receptors incubated with the corresponding ligand receptors showed no statistic differences (see Supplementary Table S2 for the number of analyzed trajectories and cells). (**c**) Monomer / dimer ratios calculated from fitted fluorescence intensity distributions with a mixed Gaussian model of Alexa546- labeled SNAP-D_{2L} receptors expressed in CHO cells with a lower expression level (0.52 ± 0.039 spots μm^{-2}) incubated with monovalent (**1a**), bivalent (**2a**) ligands compared with ligand-free SNAP-D_{2L} receptors (ctl.) of *n* analyzed cells (*n* = 15 for **1a**, 14 for **2a** and 10 for ctl.). Data in **b** and **c** represent mean ± s.d.. Statistical significance of differences in the monomer levels was determined by an unpaired *t-test* (*****p*-value < 0.0001, n.s.- not significant when compared to ctl. or as indicated by the bar).

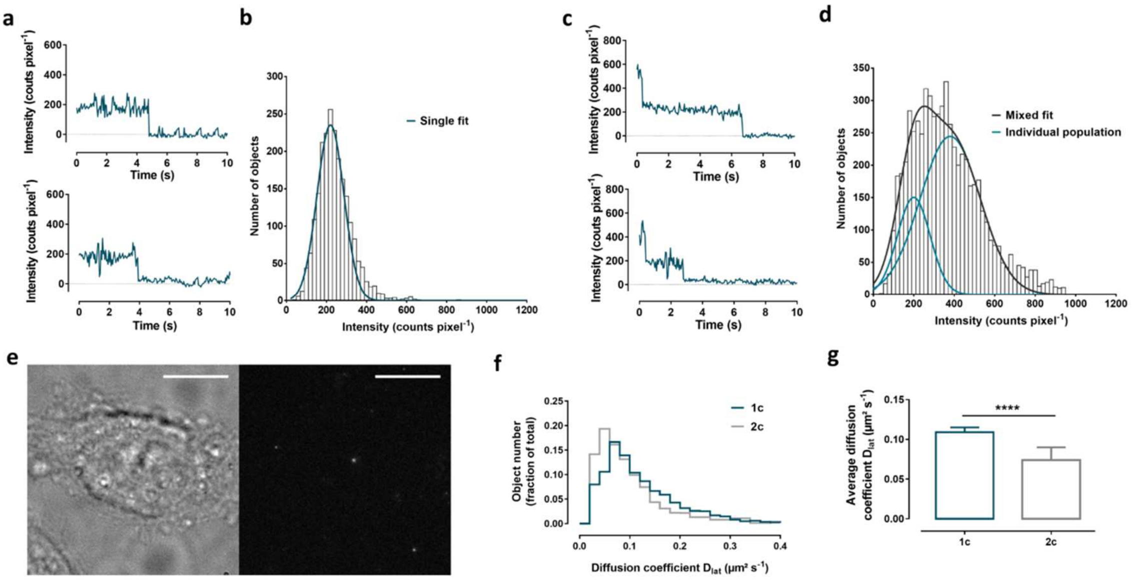

Supplementary Fig. S7| Characterization of single fluorescent monovalent (1c) and bivalent (2c) ligands by TIRF-M adhered to glass slides (a-d) and dopamine D_{2L} receptor diffusion labeled with 1c and 2c, respectively (f,g). Representative time traces of the fluorescent ligand **1c** (**a**) showed one-step photobleaching and those of **2c** showed two-step photobleaching (**c**). (**b**) Distribution of the intensity of single diffraction limited spots of **1c**. Data were fitted with a single Gaussian model. (**d**) Distribution of the fluorescence intensity of single diffraction limited spots of **2c**. Data were fitted with a mixed Gaussian model. (**e**) Validation of receptor specific dopamine D_{2L} receptor labeling by the fluorescent monovalent ligand **1c**. Representative brightfield (left) and TIRF-M image (right) of a cell stably transfected with D_{2L}, preincubated with 10 µM spiperone and labeled with **1c** (300 nM). No cell-surface staining was present. (**f**) Distribution of the diffusion coefficients (Dlat of the receptor ligand complexes D_{2L}-**1c** and D_{2L}-**2c**. (**g**) Average diffusion coefficients of the receptor-ligand complexes, D_{2L}-**1c** and D_{2L}-**2c**. The difference is statistically significant and was determined by an unpaired *t*-test (*****p*-value< 0.0001) and provides evidence for increased receptor dimerization for D_{2L} receptors labeled with the bivalent ligand **2c**. Data are mean ± s.d..

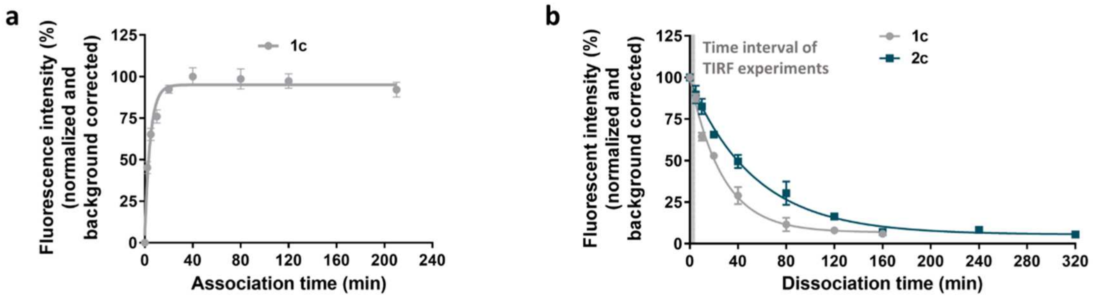

Supplementary Fig. S8 | Live cell association and dissociation kinetics of the fluorescent monovalent ligand 1c and bivalent ligand 2c. (**a**) Representative association curve of **1c** (300 nM) binding to the SNAP-D_{2L} receptor at 37°C. The binding rapidly rises to an equilibrium state. Each data point represents background corrected mean fluorescent intensity of 18 to 30 cells (mean ± s.d.) per condition and normalization to the maximum fluorescence (100 %) of this data set. The data were analyzed with a one-phase exponential association fit, giving an on-rate of 0.204 ± 0.026 min^{-1} (mean ± s.e.m). Based on this observation, the ligand incubation time for TIRF experiments was selected as 60 min at 37 °C. (**b**) Dissociation curve of the fluorescent ligands **1c** and **2c** from the SNAP-D_{2L} receptor. CHO cells stably expressing the SNAP-D_{2L} receptor were preincubated for 1 h at 37°C with **1c** (300 nM) and **2c** (1.5 µM), respectively, washed and further incubated for the indicated time periods at 23°C in the presence of 5 x 10^{-6} M spiperone in imaging buffer. The data were analyzed with a one-phase exponential decay function giving an off-rate 0.036 ± 0.0013min^{-1} for **1c** (half- life time $t_{1/2}$ of 19.2 ± 0.6 min (mean ± s.e.m)) and 0.018 ± 0.0018 min^{-1} for **2c** (half- life time $t_{1/2}$ of 38.2 ± 2.0 min (mean ± s.e.m)). Data points represent mean fluorescent intensity (mean ± s.e.m) of three independent experiments background corrected and normalized to the maximum fluorescence (100%) of each data set. The dissociation rates were determined to verify that during the image acquisition the dissociation of the monovalent and bivalent ligands was negligibly small at 24°C (shaded grey area).

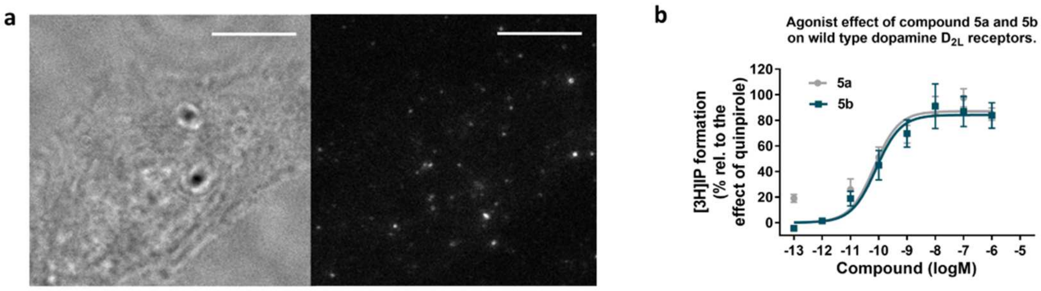

Supplementary Fig. S9 | Validation of receptor specific dopamine D_{2L} receptor labeling with the fluorescent antagonist 4a (a) and functional characterization of the fluorescent agonists 5a and 5b (b). (**a**) Validation of receptor specific dopamine D_{2L} receptor labeling with the fluorescent antagonist **4a**. Representative brightfield (left) TIRF-M image (right) of a cell stably transfected with D_{2L}, preincubated with 10 µM spiperone and labeled with fluorescent antagonist **4a** (labeling concentration 10-fold K_i-value of the corresponding ligand). No cell-surface staining is present. (**b**) IP-accumulation assay for activation of wild-type (wt) dopamine D_{2L} receptors. The investigation of activation of the wt D_{2L} by compounds **5a** and **5b** reveals agonist activity relative to the effect of the reference agonist quinpirole. The representative curves from three individual experiments give potencies of 0.07 nM and 0.08 nM and in estimated intrinsic activities of ca. 87 % and 84 % for **5a** and **5b**, respectively. Points indicate means ± s.e.m. of three independent experiments, each performed in triplicate. Experiments were carried out as described in the literature [4].

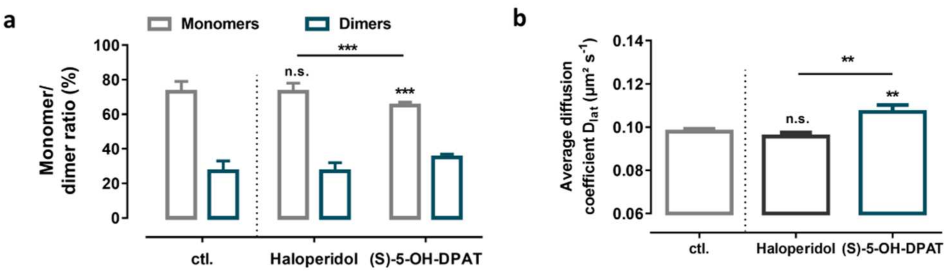

Supplementary Fig. S10. | Influence of the antagonist haloperidol and the agonist (S)-5-OH-DPAT on SNAP-D_{2L} receptor dimerization and diffusion. (**a**) Monomer / dimer ratios calculated from fitted fluorescence intensity distributions with a mixed Gaussian model of Alexa546-labeled SNAP-D_{2L} receptors (ctl.) (receptor density level: ++ 0.52 ± 0.039 spots μm^{-2} (mean ± s.d., 10 cells) incubated with dopamine receptor antagonist haloperidol (9 cells) and agonist (S)-5-OH-DPAT (8 cells) and their average diffusion coefficient (D_{lat}) in (**b**). Data represent mean ± s.d.. Statistical analyses were performed by an unpaired t-test (****p-value*** < 0.01, ******p-value*** < 0.001, n.s - not significant when compared to ctl. or as indicated by the bar).

Supplementary Table S1. Receptor binding data for the monovalent, bivalent, and control ligands **1a-c**, **2a-c**, **3a,b**, **4a,b** and **5a,b** at human D_{2L}, D_{2S} and D_3 and SNAP-D_{2L} receptors.

	K_i ± s.d. (nM)[a]			
	[^{3}H]spiperone			
**Ligand**	**hD_{2L}**	**hD_{2S}**	**hD_3**	**SNAP-D_{2L}**
**1a**	67 ± 33	53 ± 37	615 ± 370	280 ± 190
**1b**	15 ± 8.0	18 ± 5.7	70 ± 24	4.8 ± 0.28
**1c**	30 ± 4.2	27 ± 2.1	44 ± 2.1	n.d.
**2a**	22 ± 6.7	41 ± 19	84 ± 24	20 ± 7.1
**2b**	3.0 ± 0.29	3.0 ± 0.92	2.6 ± 0.64	2.4 ± 1.1
**2c**	150 ± 39	110 ± 63	56 ± 32	n.d.
**3a**	9.8 ± 9.8	7.3 ± 1.3	220± 83	n.d.
**3b**	19 ± 8.2	17 ± 8.1	100 ± 33	n.d.
**4a**	3.8 ± 2.8	7.8 ± 3.7	5.3 ± 2.3	n.d.
**4b**	29 ± 9.6	42 ± 12	19 ± 4.3	n.d.
**5a**	8.8 ± 6.1	7.1 ± 2.1	0.44 ± 0.20	n.d.
**5b**	6.0 ± 2.7	7.7 ± 5.	2.8 ± 0.45	n.d.
**Haloperidol**	1.1 ± 6.1	0.75 ± 0.27.	7.50 ± 5.0.	n.d.
**S-(–)-5-OH-DPAT**	8.6 ± 6.5	7.1 ± 2.0.	1.3 ± 0.40.	n.d.

[a]*K_i values in nM ± standard deviation (s.d.) derived from at least two individual experiments, each performed in triplicate.*

Supplementary Table S2. Summary of the analysis of labeled SNAP-D_{2L} and D_{2L} receptor mobilities and dimerization with bound monovalent (**1a-c**), bivalent (**2a-c**) and control (**3a/b**) ligands.

	Ligand	Receptor	Density (spots µm^{-2})	Average D_{lat} 24°C (µm² s^{-1})	Number of analyzed trajectories	Monomer/ dimer ratio (%)	Number of analyzed cells
	**no**	SNAP-D_{2L}	0.67 ± 0.03	0.084± 0.006 (0.078 – 0.090)	12031	70/30 ± 4	10
**10 x K_i value labeling concentration**	**1a**	SNAP-D_{2L}	0.68 ± 0.04	0.087 ± 0.003 (0.085 - 0.089)	17983	70/30 ± 3	16
	**2a**	SNAP-D_{2L}	0.65 ± 0.04	0.074 ± 0.004 (0.070 - 0.078)	8741	34/66 ± 4	8
	**1b**	SNAP-D_{2L}	0.66 ± 0.04	0.085± 0.004 (0.082 - 0.087)	7535	71/29 ± 2	8
	**2b**	SNAP-D_{2L}	0.64 ± 0.04	0.075 ± 0.004 (0.072 - 0.079)	17822	42/58 ± 3	16
	**3a**	SNAP-D_{2L}	0.67 ± 0.03	0.083 ± 0.004 (0.079 - 0.088)	6781	67/33 ± 5	8
	**3b**	SNAP-D_{2L}	0.68 ± 0.04	0.083 ± 0.005 (0.079 - 0.084)	8682	66/34 ± 4	10
	**1c**	SNAP-D_{2L}	0.66 ± 0.04	0.080 ± 0.004 (0.077 - 0.084)	10749	74/26 ± 4	9
	**1c**	D_{2L}	0.64 ± 0.04	0.109 ± 0.006 (0.098 – 0.112)	10515	71/29 ± 2	6
	**2c**	D_{2L}	0.66 ± 0.04	0.074 ± 0.016 (0.070 - 0.079)	9610	n.d	6
**100 x K_i value labeling concentration**	**1a**	SNAP-D_{2L}	0.69 ± 0.03	0.083 ± 0.005 (0.079 - 0.087)	8087	70/30 ± 3	9
	**2a**	SNAP-D_{2L}	0.65 ± 0.04	0.075 ± 0.005 (0.073 - 0.079)	10062	34/66 ± 2	12
	**1b**	SNAP-D_{2L}	0.69 ± 0.04	0.081 ± 0.006 (0.076 - 0.084)	10441	71/29 ± 3	10
	**2b**	SNAP-D_{2L}	0.67 ± 0.02	0.071 ± 0.05 (0.067 - 0.074)	8357	43/56 ± 4	9

Density: number of objects (mean ± s.d.) identified at the beginning of the records. *Average D_{lat}:* average lateral diffusion coefficient (mean ± s.d.) (95 % confidence interval), (slow-moving objects D_{lat} < 0.02 µm² s^{-1} excluded). *No. of spots:* number of objects tracked during the whole record (slow-moving objects D_{lat} < 0.02 µm² s^{-1} excluded). *Monomer/ dimer ratio*: calculated from a fluorescent spot intensity distribution fitted with a mixed Gaussian model (sum of two Gaussian functions) (mean ± s.d.).

Supplementary Table S3. Summary of the analysis of labeled monomeric SNAP-CD86 and dimeric SNAP-CD28 receptor mobilities.

Protein	Density (spots µm^{-2})	Average D_{lat} 24°C (µm² s^{-1})	Number of analyzed trajectories	Number of analyzed cells
SNAP-CD86	0.64 ± 0.04	0.118 ± 0.006 (0.096 - 0.122)	6603	9
SNAP-CD28	0.67 ± 0.03	0.104 ± 0.008 (0.095 – 0.116)	3999	7

Density: number of objects (mean ± s.d.) identified at the beginning of the records. *Average D_{lat}:* average lateral diffusion coefficient (mean ± s.d.) (95 % confidence interval), (slow-moving objects D_{lat} < 0.02 µm² s^{-1} excluded). *No. of spots:* number of objects tracked during the whole record (slow-moving objects D_{lat} < 0.02 µm² s^{-1} excluded).

Supplementary Table S4. Photobleaching kinetics of D_{2L} receptor ligand complexes with bound fluorescent antagonists (**4a,b**) and agonists (**5a,b**) under TIRF illumination.

**Receptor**	**Ligand**	**Photobleaching kinetics**		
		**k (s^{-1})**	**$t_{1/2}$ (s)**	**Number of analyzed cells**
**hD_{2L}**	**4a**	0.112 ± 0.004 (0.108 - 0.123)	6.10 ± 0.24 (5.63 - 6.67)	6
	**4b**	0.089 ± 0.013 (0.080 - 0.098)	7.90 ± 0.31 (7.20 - 8.60)	11
	**5a**	0.119 ± 0.006 (0.104 - 0.133)	6.00 ± 0.33 (0.104 - 0.133)	8
	**5b**	0.089 ± 0.022 (0.074 - 0.105)	8.13 ± 1.13 (6.85 - 9.40)	8

Mean values ± standard deviation (s.d.) (95 % confidence interval) are derived from at least three independent experiments.

Supplementary Table S5. Summary of the analysis of D_{2L}, D_{2S} and D_3 receptor mobilities and dimerization when complexed with the fluorescent antagonists (**4a,4b**) and agonists (**5a,5b**).

Ligand	Receptor	Density (spots µm^{-2})	Average D_{lat} 24°C (µm² s^{-1})	No. of spots	Monomer/ dimer ratio (%)	Number of analyzed cells
**4a**	D_{2L} wt	0.69 ± 0.03	0,100 ± 0.009 (0.092– 0.108)	14983	70 / 30 ± 3	18
**5a**	D_{2L} wt	0.65 ± 0.04	0.118 ± 0.005 (0.116 – 0.123)	15154	55/45 ± 4	19
**4a**	D_{2s} wt	0.65 ± 0.05	0.105 ± 0.002 (0.099 – 0.111)	10822	82/18 ± 3	13
**5a**	D_{2s} wt	0.66 ± 0.02	0.128 ± 0.003 (0.117 - 0.139)	9535	71/29 ± 4	12
**4a**	D_3 wt	0.69 ± 0.03	0.114 ± 0.008 (0.108 – 0.119)	9781	90/10 ± 4	12
**5a**	D_3 wt	0.67 ± 0.03	0.119 ± 0.006 (0.114 - 0.124)	11682	74/26 ± 2	13
**4b**	D_{2L} wt	0.69 ± 0.04	0.104 ± 0.006 (0.095 – 0.116)	13350	71/29 ± 2	14
**5b**	D_{2L} wt	0.67 ± 0.04	0.120 ± 0.021 (0.109 – 0.128)	10907	58/42 ± 2	17
**4b**	D_{2s} wt	0.69 ± 0.01	0.110 ± 0.008 (0.102 – 0.125)	17365	81/19 ± 3	19
**5b**	D_{2s} wt	0.65 ± 0.04	0.130 ± 0.003 (0.109 - 0.17)	8856	64/36 ± 4	12
**4b**	D_3 wt	0.69 ± 0.04	0.116 ± 0.003 (0.108 – 0.120)	11024	91/9 ± 3	15
**5b**	D_3 wt	0.66 ± 0.02	0.121 ± 0.006 (0.111 - 0.132)	7812	75/25 ± 3	10

Density: number of objects (mean ± s.d.) identified at the beginning of the records. *Average D_{lat}:* average lateral diffusion coefficient (mean ± s.d.) (95 % confidence interval), (slow-moving objects D_{lat} < 0.02 µm² s^{-1} excluded). *No. of spots:* number of objects tracked during the whole record (slow-moving objects D_{lat} < 0.02 µm² s^{-1} excluded). *Monomer/dimer ratio*: calculated from a fluorescent spot intensity distribution fitted with a mixed Gaussian model (sum of two Gaussian functions) (mean ± s.d.).

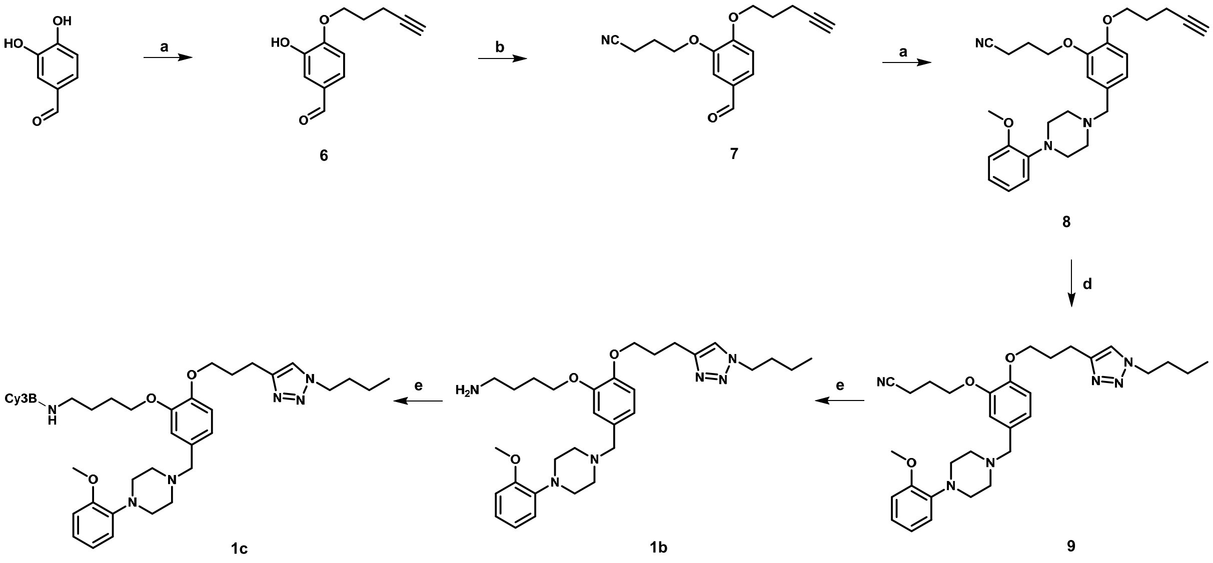

Supplementary Fig. S11 | Synthesis of the monovalent ligand 1b and its fluorescent analog 1c. Reagents and conditions: (a) Diethylazadicarboxylate, triphenylphosphine, 4-pent-yn-1-ol, THF, 0 °C-rt, 16 h, 45 %; (b) 4-Bromobutanenitrile, K_2CO_3, KI, CH_3CN, 90 °C, 16 h, 88 %; (c) 1-(2-Methoxyphenyl)piperazine, $Na(OAc)_3BH$, CH_2Cl_2, 0 °C-rt, 16 h, 77 %; (d) azidobutane, $CuSO_4.5H_2O$, sodium ascorbate, *t*-BuOH/H_2O/CH_2Cl_2, rt, 16 h, 95 %; (e) $LiAlH_4$, THF, 0 °C-rt, 1 h, 50 %; (f) Cy3B-NHS ester, DIPEA, DMF, rt, 3 h, 90 %.

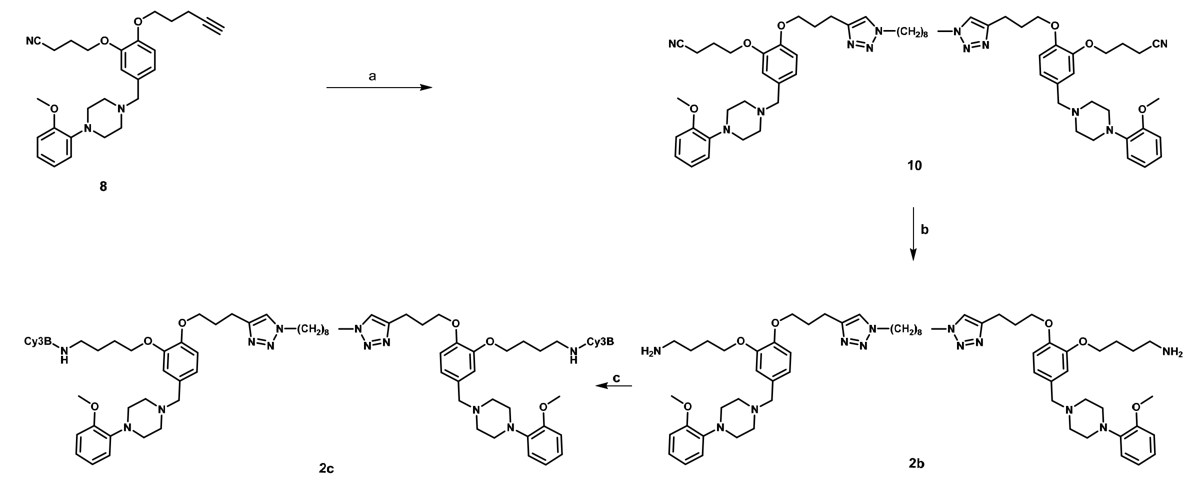

Supplementary Fig. S12 | Synthesis of the bivalent ligand 2b and its fluorescent analog 2c. Reagents and conditions: (a) 1,8 diazidooctane, $CuSO_4.5H_2O$, sodium ascorbate, t-BuOH/H_2O/CH_2Cl_2, rt, 16 h, 95 %; (b) $LiAlH_4$, THF, 0 °C-rt, 1 h, 25 %; (c) Cy3B-NHS ester, DIPEA, DMF, rt, 3 h, 92 %.

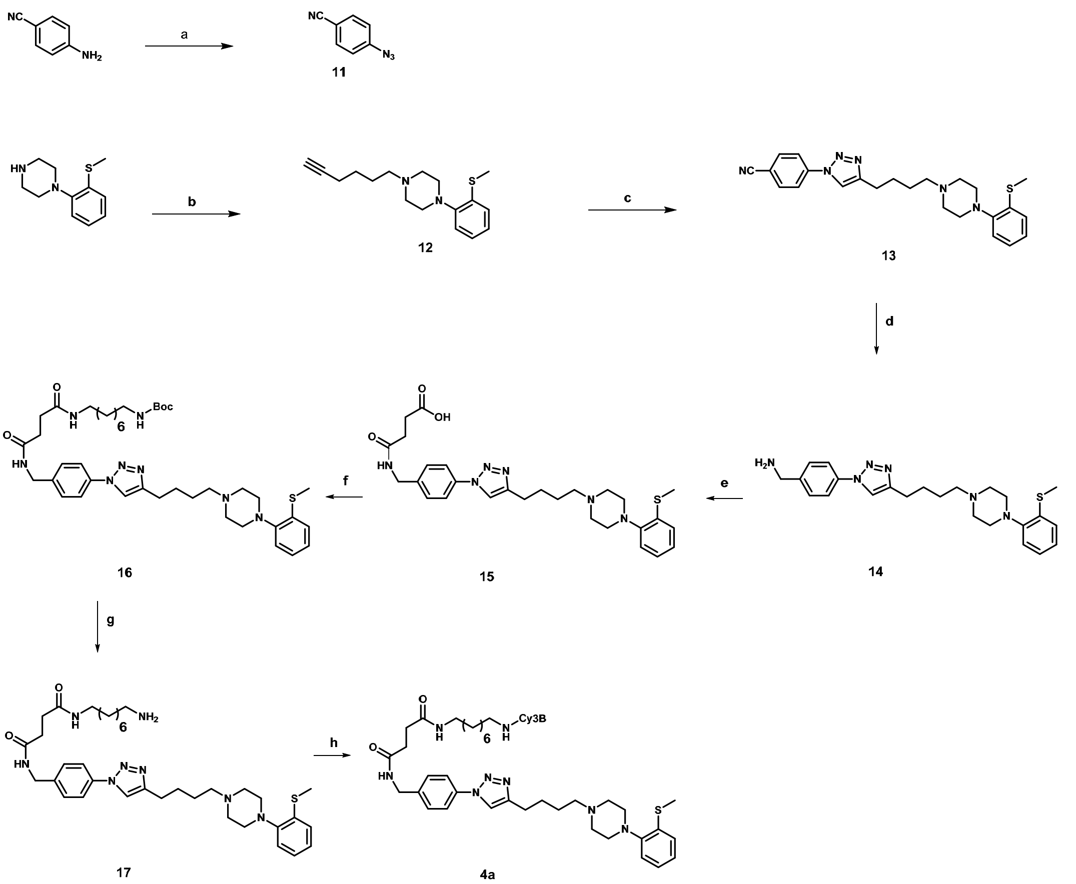

Supplementary Fig. S13 | Synthesis of the fluorescent antagonist 4a. Reagents and conditions: (a) conc. HCl, $NaNO_2$, NaN_3, 0 °C, 3 h; (b) 6-Chlorohexyne, K_2CO_3, KI, CH_3CN, 90 °C, 16 h; (c) 4-Azidobenzonitrile **11**, $CuSO_4.5H_2O$, sodium ascorbate, t-BuOH-H_2O-CH_2Cl_2, rt, 16 h; (d) $LiAlH_4$, THF, 0 °C-rt, 2 h; (e) Succinic anhydride, NEt_3, DMF, microwave, 100 °C, 1 h; (f) Tert-butyl(8-aminooctyl) carbamate, TBTU, DIPEA, 0 °C-rt, 16 h; (g) 4M HCl in dioxane, rt, 16 h; (h) Cy3B-NHS ester, DIPEA, DMF, rt, 24 h.

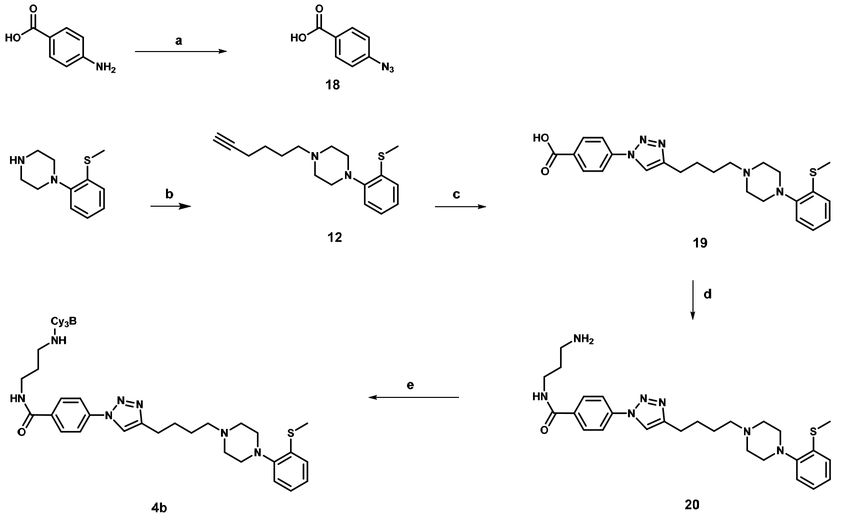

Supplementary Fig. S14| Synthesis of the fluorescent antagonist 4b. Reagents and conditions: (a) conc. HCl, $NaNO_2$, NaN_3, 0 °C, 3 h; (b) 6-Chlorohexyne, K_2CO_3, KI, CH_3CN, 90 °C, 16 h; (c) 4-Azidobenzoic acid **18**, $CuSO_4.5H_2O$, sodium ascorbate, t-BuOH-H_2O-CH_2Cl_2, rt, 16 h, 90 %; (d) 1,3-Diaminopropane, TBTU, DIPEA, DMF, 0 °C-rt, 16 h, 40 %; (e) Cy3B-NHS ester, DIPEA, DMF, rt, 3 h, 89 %.

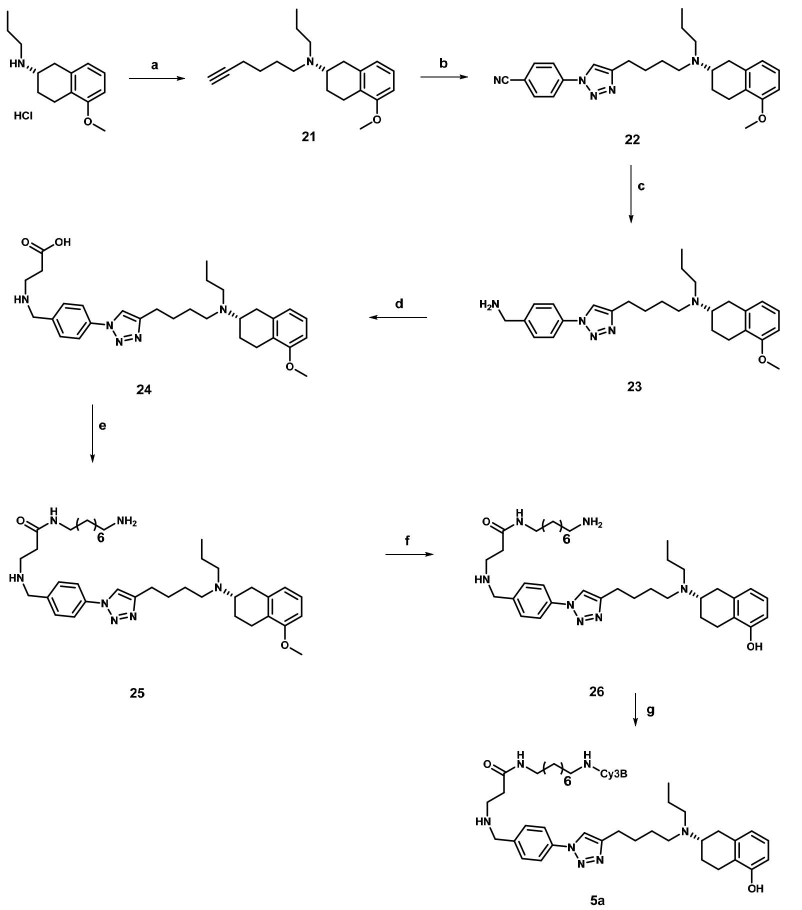

Supplementary Fig. S15 | Synthesis of the fluorescent antagonist 5a. Reagents and conditions: (a) 6-Chlorohexyne, K_2CO_3, KI, CH_3CN, 90 °C, 16 h; (b) 4-Azidobenzonitrile **11**, $CuSO_4.5H_2O$, sodium ascorbate, t-BuOH-H_2O-CH_2Cl_2, rt, 16 h, 62 %; (c) $LiAlH_4$, THF, 0 °C-rt, 2 h; (d) Succinic anhydride, NEt_3, DMF, microwave, 100 °C, 1 h, 75 %; (e) 1,8-Diaminooctane, TBTU, DIPEA, 0 °C-rt, 16 h, 40 %; (f) BBr_3, CH_2Cl_2, -40 °C-rt, 16 h, 52 %; (g) Cy3B-NHS ester, DIPEA, DMF, rt, 3 h, 92 %.

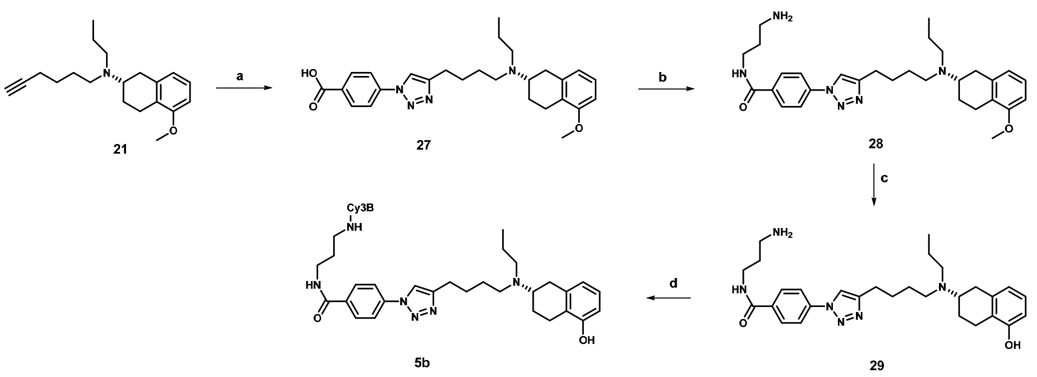

Supplementary Fig. S16 | Synthesis of the fluorescent antagonist 5b. Reagents and conditions: (a) 1, $CuSO_4.5H_2O$, sodium ascorbate, t-BuOH-H_2O-CH_2Cl_2, rt, 16 h, 60 %; (b) 1,3-Diaminopropane, TBTU, DIPEA, DMF, 0 °C-rt, 16 h, 90 %; (c) BBr3, CH2Cl2, -40 °C-rt, 16 h, 84 %; (d) Cy3B-NHS ester, DIPEA, DMF, rt, 3 h, 89 %.

Supplementary Note S4: Organic synthesis.

General methods. All reactions requiring anhydrous conditions were carried out under nitrogen and the solvents were dried appropriately before use. All reactions were monitored by TLC using Kieselgel 60 F_{254} plates. Visualization of the reaction components was achieved using UV fluorescence (254 nm) and $KMnO_4$ stain. Silica gel chromatography was carried out over Silicagel 60. The yields reported are after purification. Cy3B N-hydroxysuccinimide (NHS) ester was purchased from Amersham Biosciences. ^{1}H, ^{13}C NMR spectra were recorded in deuterated solvents and chemical shifts (δ) are quoted in parts per million (ppm) calibrated to TMS (^{1}H and ^{13}C). Coupling constants (J) are measured in Hertz (Hz). The following abbreviations are used to describe multiplicities: s = singlet, d = doublet, t = triplet, q = quartet, b = broad, m = multiplet. Purity and identity were assessed by analytical RP-HPLC (Agilent 1100 analytical series, column: Zorbax Eclipse XDB-C8 analytical column, 4.6 × 150 mm, 5 µm, flow rate: 0.5 ml/min, detection wavelength: 254 nm) coupled to a Bruker Esquire 2000 mass detector equipped with an ESI- or APCI-trap. Purities of the products were assessed from HPLC-MS using MeOH / H_2O 0.1 % HCOOH as the solvent system and employing the following gradient system 0-3 min: 10 % MeOH, 3-18 min: 10-100 % MeOH, 18- 24 min: 100 % MeOH, 24-30 min: 100-10 % MeOH.

General procedure. *General procedure I (GP I): Representative experimental procedure for the synthesis of terminal alkynes.*To a mixture of secondary amines (1-phenylpiperizines or aminoindane), K_2CO_3 (2 eq) and KI (1 eq) in CH_3CN was added the corresponding haloalkynes at room temperature and the reaction mixture was allowed to reflux for overnight. The reaction was quenched by the addition of H_2O and the aqueous phase was extracted with DCM (3 times). The combined organic layers were dried over Na_2SO_4 and evaporated under reduced pressure to give the crude compound.

General procedure II (GP II): Representative experimental procedure for the synthesis of aromatic azides. To an ice cooled solution of 4-substituted aniline (1 eq) in conc. HCl, aqueous solution of $NaNO_2$ (1 eq) was added slowly. After 30 mins, a solution of NaN_3 (10 eq) was added at 0 º C. After an incubation of 1-2 hour at room temperature, the product was extracted (3 times) with EtOAc. The organic phase was dried over anhydrous Na_2SO_4 and concentrated in vacuo to obtain the corresponding aromatic azides in the pure form.

General procedure III (GP III): Representative experimental procedure for the Cu(II) catalyzed 1,3-cycloaddition. To the mixture of corresponding alkynes and aromatic/aliphatic azides in a solvent system of *t*-BuOH-H_2O-DCM (1:1:1) was added $CuSO_4 \cdot 5H_2O$ (5 mol %) and Na-Ascorbate (10 mol %) and was stirred at room temperature overnight. After the completion of the reaction (monitored by TLC), it was quenched by the addition of 0.1 M EDTA solution. The organic compounds were extracted

with DCM (3 times), dried over Na_2SO_4 and evaporated. The pure compound was isolated by silica-gel column chromatography.

*General procedure IV (GP IV): Representative experimental procedure for the solution phase amide coupling.*To a solution of the benzoic acid derivatives and DIPEA in DCM at 0 ° C was added TBTU in anhydrous DMF and the mixture was stirred for 30 min. Then it was added to a solution of primary amine (10 eq) in DMF and stirred for 16 hours at room temperature. The reaction was quenched by the addition of satd. $NaHCO_3$, extracted with DCM (2 times) and the combined organic phases were dried over anhydrous Na_2SO_4 and concentrated in vacuo to obtain the crude product. The pure compound was isolated by a short silica-gel column chromatography.

General procedure V (GP V): Representative experimental procedure for preparation of Cy3B conjugates. Aliquots of a solution of the primary amines in dry dimethylformamide (DMF) and diisopropylethylamine (DIPEA) were added to a vial of Cy3B N-hydroxysuccinimide (NHS) ester. The vial was kept at room temperature for 3 h. Reverse-phase HPLC purifications yielded the pure compounds.

General procedure VI (GP VI): Representative experimental procedure for reductive amination reaction. The aldehyde was dissolved in dichloromethane and cooled down to 0 °C. To this a solution of the corresponding amine in dichloromethane was added followed by $Na(OAc)_3BH$ and stirred at 0 °C for 1 h. It was then warmed up to room temperature and stirred at room temperature for 16 h. The reaction was quenched with a saturated solution of $NaHCO_3$ and extracted three times with dichloromethane. The combined organic phases was dried over anhydrous Na_2SO_4 and concentrated in vacuo to obtain the crude product.

General procedure VII (GP VII): Representative experimental procedure for oxime formation. A solution of hydroxylamine hydrochloride in water and 2N NaOH solution was cooled down to 0 °C and the pH was adjusted to 5 with 2N HCl. To this a solution of the carbaldehyde in ethanol was added drop wise and refluxed for 2 hours. The reaction was quenched with a saturated solution of $NaHCO_3$ and extracted three times with dichloromethane. The combined organic phases was dried over anhydrous Na_2SO_4 and concentrated in vacuo to obtain the crude product.

Preparative HPLC

Preparative RP-HPLC was performed using Agilent 1100 preparative series.

Method A

Column: Nucleodur C18 HTec, 32 × 250 mm, 5 µm particles, flow rate 32 mL /min, detection wavelengths: 220, 254 nm)

Solvent: Acetonitrile (ACN), water 0.1 % TFA

Gradient: 5-50 % ACN in 0-20 min, 50-95 % ACN in 20-22 min, 95-95 % ACN in 22-25 min, 95-5 % ACN in 25-30 min.

Method B

Column: Nucleodur C18 HTec, 10 × 250 mm, 5 µm particles, flow rate 4 mL /min, detection wavelengths: 220, 254, 565 nm)

Solvent: Acetonitrile (ACN), water 0.1 % TFA

Gradient: 5-50 % ACN in 0-20 min, 50-95 % ACN in 20-22 min, 95-95 % ACN in 22-25 min, 95-5 % ACN in 25-30 min.

Method C

Column: Nucleodur C18 HTec, 32 × 250 mm, 5 µm particles, flow rate 32 mL /min, detection wavelengths: 220, 254 nm)

Solvent: Acetonitrile (ACN), water 0.1 % HCOOH

Gradient: 3-50 % ACN in 0-20 min, 50-95 % ACN in 20-21 min, 95-95 % ACN in 21-24 min, 95-3 % ACN in 24-26 min, 3-3 % ACN in 26-30 min.

Method D

Column: Nucleodur C18 HTec, 32 × 250 mm, 5 µm particles, flow rate 32 mL /min, detection wavelengths: 220, 254 nm)

Solvent: Acetonitrile (ACN), water 0.1 % TFA

Gradient: 5-50 % ACN in 0-15 min, 50-100 % ACN in 15-16 min, 100-100 % ACN in 16-18 min, 100-5 % ACN in 18-19 min, 5-5 % ACN in 19-22 min.

Method E

Column: Nucleodur C18 HTec, 10 × 250 mm, 5 µm particles, flow rate 4 mL /min, detection wavelengths: 220, 254, 565 nm)

Solvent: Acetonitrile (ACN), water 0.1 % TFA

Gradient: 5-95 % ACN in 0-20 min, 95-95 % ACN in 20-23 min, 95-5 % ACN in 23-26 min.

3-Hydroxy-4-(pent-4-yn-1-yloxy)benzaldehyde (6)

3,4-dihydroxybenzaldehyde (0.2 g, 1.4 mmol), 5-chloropent-1-yne (0.15 mL, 1.4 mmol), K_2CO_3 (0.2 g, 1.4 mmol) and KI (0.24 g, 1.4 mmol) were mixed together in acetone 5 mLand heated for 24 h at 60 °C.

The reaction was quenched with water and extracted three times with ethylacetate. The organic layer was collected and dried with Na_2SO_4 and concentrated in vacuo. Flash chromatographic purification using 8:2 hexane ethylacetate yielded the title compound (80 mg) in 28 % yield as colourless oil. ^{1}H-NMR (600 MHz, $CDCl_3$): δ (ppm) 2.02 (t, *J* = 2.7 Hz, 1H), 2.08 (pent, *J* = 6.3 Hz, 2H), 2.42 (dt, *J* = 6.7, 2.7 Hz, 2H), 4.24 (t, *J* = 6.3 Hz, 2H), 5.81 (s, 1H), 6.96 (d, *J* = 8.08 Hz, 1H), 7.40 (dd, *J* = 8.3, 2.0 Hz, 1H), 7.43 (d, *J* = 2.0 Hz, 1H), 9.83 (s, 1H). ^{13}C-NMR (150 MHz, CD_3OD): δ (ppm) 15.5, 27.7, 67.9, 69.4, 83.2, 110.9, 114.3, 124.3, 130.7, 146.3, 150.9, 190.9.

4-(5-Formyl-2-(pent-4-yn-1-yloxy)phenoxy)butanenitrile (7)

Compound **6** (30 mg, 0.15 mmol), 4-Bromobutanenitrile (0.03 mL, 0.3 mmol), K_2CO_3 (41 mg, 0.3 mmol) and KI (50 mg, 0.3 mmol) were mixed together in acetonitrile 5 mL and heated for 16 h at 100 °C. The reaction was quenched with water and extracted three times with ethylacetate. The organic layer was collected and dried with Na_2SO_4 and concentrated in vacuo. Flash chromatographic purification using 6:4 hexane ethylacetate yielded the title compound (35 mg) in 88 % yield as colourless oil. ^{1}H-NMR (600 MHz, $CDCl_3$): δ (ppm) 2.02 (t, *J* = 2.7 Hz, 1H), 2.08 (pent, *J* = 6.3 Hz, 2H), 2.19-2.23 (m, 2H), 2.45 (dt, *J* = 6.7, 2.7 Hz, 2H), 2.64 (t, *J* = 7.2 Hz, 2H), 4.17 (t, *J* = 5.7 Hz, 2H), 4.21 (*t*, *J* = 6.1 Hz, 2H), 7.02 (d, *J* = 8.08 Hz, 1H), 7.42 (d, *J* = 2.0 Hz, 1H), 7.49 (dd, *J* = 8.3, 2.0 Hz, 1H), 9.85 (s, 1H). ^{13}C-NMR (150 MHz, CD_3OD): δ (ppm) 14.1, 15.0, 25.5, 27.9, 66.6, 67.2, 69.2, 82.9, 111.8, 112.1, 119.1, 127.2, 130.1, 148.6, 154.4, 190.6.

4-(5-((4-(2-Methoxyphenyl)piperazin-1-yl)methyl)-2-(pent-4-yn-1-yloxy)phenoxy) butanenitrile (8)

Following GP VI from **7** (25 mg, 0.092 mmol), 1-(2-methoxyphenyl)piperazine (26 mg, 0.14 mmol) and sodium triacetoxy borohydride (29 mg, 0.14 mmol) compound **8** was synthesised. Flash chromatographic purification with ethylacetate yielded the title compound (32 mg) in 77 % yield as yellow oil. ^{1}H-NMR (600 MHz, $CDCl_3$): δ (ppm) 1.98 (t, *J* = 2.7 Hz, 1H), 2.03 (pent, *J* = 6.6 Hz, 2H), 2.13-2.17 (m, 2H), 2.41-2.44 (m, 2H), 2.62-2.65 (m, 6H), 3.09 (bs, 4H), 3.51 (s, 2H), 3.85 (s, 3H), 4.08-4.13 (m 4H), 6.84-6.87 (m, 1H), 6.88-6.95 (m, 5H), 6.97-7.00 (m, 1H). ^{13}C-NMR (150 MHz, $CDCl_3$): δ (ppm) 14.2, 15.2, 25.7, 28.2, 50.6, 53.3, 55.3, 62.7, 65.0, 66.9, 68.9, 83.4, 111.2, 113.8, 114.0, 115.9, 118.2, 119.4, 120.6, 120.9, 122.8, 134.2, 141.4, 148.2, 152.3. LC-MS-ESI**:** *m/z* Calcd. for $C_{27}H_{33}N_3O_3$: 448.2, $[M+H]^+$: *m/z* Found: 448.5 $[M+H]^+$, purity LC-MS 90 %, t_R = 16.6 min.

4-(2-(3-(1-Butyl-1H-1,2,3-triazol-4-yl)propoxy)-5-((4-(2-methoxyphenyl)piperazin-1-yl)methyl) phenoxy)butanenitrile (9)

Compound **8** (57 mg, 0.13 mmol), was dissolved in a mixture of water, methanol, dichloromethane 1:1:1 and azidobutane (25 mg, 0.25 mmol) added followed by $CuSO_4.5H_2O$ (3 mg, 0.007 mmol) and sodium ascorbate (7.5 mg, 0.04 mmol) and it was stirred for 16 h at room temperature. Then 2 mL, 0.1 M EDTA solution was added and the organic layer was extracted three times with dichloromethane and dried with $NaSO_4$ and concentrated in vacuo. Flash chromatographic purification using Methanol Ethylacetate 20 % and 1 % ammonia yielded the title compound (60 mg, 86 %) as colourless oil. ^{1}H-NMR (360 MHz, $CDCl_3$): δ (ppm) 0.94 (t, *J* = 7.3 Hz, 3H), 1.34 (sextet, *J* = 7.3 Hz, 2H), 1.86 (quint, *J* = 7.1 Hz, 2H), 2.12-2.25 (m, 4H), 2.63-2.69 (m, 6H), 2.92 (t, *J* = 7.7 Hz, 2H), 3.12 (bs, 4H), 3.55 (bs, 2H), 3.85 (s, 3H), 4.04 (t, *J* = 6.2 Hz, 2H), 4.14 (t, *J* = 6.2 Hz, 2H), 4.31 (t, *J* = 7.3 Hz, 2H), 6.82-6.93 (m, 5H), 6.95-7.02 (m, 2H), 7.31 (s, 1H). ^{13}C-NMR (90 MHz, $CDCl_3$): δ (ppm) 13.5, 14.1, 19.7, 22.2, 25.7, 29.0, 32.3, 49.9, 50.4, 53.2, 55.4, 62.6, 67.2, 68.1, 111.2, 113.5, 116.2, 118.3, 119.4, 120.8, 121.0, 122.9, 123,0, 147.0, 148.1, 152.3. LC-MS-ESI**:** *m/z* Calcd. for $C_{31}H_{42}N_6O_3$: 547.3, $[M+H]^+$: *m/z* Found: 547.7 $[M+H]^+$, purity LC-MS 95 %, t_R = 16.7 min.

4-(2-(3-(1-Butyl-1H-1,2,3-triazol-4-yl)propoxy)-5-((4-(2-methoxyphenyl)piperazin-1-yl)methyl)phenoxy)butan-1-amine (1b)

Compound **9** (60 mg, 0.011 mmol) was dissolved in 2 mL dry THF and cooled down to 0 °C. Then a 4 M solution of $LiAlH_4$ in diethylether (0.3 mL, 0.11 mmol) was added and stirred at 0 °C for 2 hours. It was then quenched with water and filtered through a bed of celite and Na_2SO_4 and washed three times with THF. Preparative HPLC purification employing method A (t_R = 16.5 min), yielded 28 mg (50 %) of **1b** as colourless oil. ^{1}H-NMR (600 MHz, $CDCl_3$): δ (ppm) 0.94 (t, *J* = 7.3 Hz, 3H), 1.32 (sextet, *J* = 7.1 Hz, 2H), 1.86 (quint, *J* = 7.1 Hz, 2H), 1.96 (bs, 4H), 2.16 (bs, 2H), 2.92 (bs, 2H), 3.13-3.25 (m, 6H), 3.50-3.59 (m, 4H), 3.86 (s, 3H), 4.04-4.07 (m, 4 H), 4.17 (bs, 2H), 4.32 (t, *J* = 6.9 Hz, 2H), 6.84-6.95 (m, 5H), 7.07-7.10 (m, 2H), 7.57 (s, 1H). ^{13}C-NMR (150 MHz, $CDCl_3$): δ (ppm) 13.3, 19.6, 21.4, 24.9, 25.9, 28.6, 31.9, 39.9, 47.6, 50.8, 51.7, 55.4, 60.9, 67.4, 68.6, 111.5, 112.5, 115.0, 119.0, 120.6, 121.2, 122.3, 124.4, 124.9, 138.2, 148.5, 149.6, 152.1, 161.4. LC-MS-ESI**:** *m/z* Calcd. for $C_{31}H_{46}N_6O_3$: 551.4, $[M+H]^+$: *m/z* Found: 551.8 $[M+H]^+$, purity LC-MS 95 %, t_R = 14.2 min. HRMS Calcd. for $C_{31}H_{47}N_6O_3$: 551.36997, $[M+H]^+$: *m/z* Found: 551.37042 $[M+H]^+$.

1b-Cy3B conjugate (1c)

Following the GP V, the reaction was performed from compound **1b** (0.7 mg, 1.3 µmol) in DMF (0.3 mL), DIPEA (1.1 µL, 6.5 µmol) and Cy3B-NHS ester (0.5 mg, 0.65 µmol), title compound **1c** was

prepared. Preparative RP-HPLC by method B, (t_R: 19.5 min) and subsequent lyophilization to afford the conjugate as the TFA salt (0.7 mg, 90 %). Purity and identity was assessed by analytical HPLC coupled to a Bruker Esquire 2000 mass detector equipped with an ESI-trap. Purity: 99% (t_r: 16.5 min), LC-MS-ESI: *m/z* Calcd. for $C_{62}H_{76}N_8O_8S$: 547.3, $[M+2H]^{2+}$: *m/z* Found: 547.9 $[M+2H]^{2+}$.

4,4'-(((((Octane-1,8-diylbis(1H-1,2,3-triazole-1,4-diyl))bis(propane-3,1-diyl))bis(oxy))bis(5-((4-(2-methoxyphenyl)piperazin-1-yl)methyl)-2,1-phenylene))bis(oxy))dibutanenitrile (10)

Compound **8** (32 mg, 0.071 mmol), was dissolved in a mixture of water, methanol dichloromethane 1:1:1 and 1,8 diazidooctane (7 mg, 0.035 mmol) added followed by $CuSO_4.5H_2O$ (2 mg, 0.007 mmol) and sodium ascorbate (4.2 mg, 0.021 mmol) and it was stirred for 16 h at room temperature. Then 2 mL, 0.1 M EDTA solution was added and the organic layer was extracted three times with dichloromethane and dried with $NaSO_4$ and concentrated in vacuo. Flash chromatographic purification using Methanol Ethylacetate 20 % and 1 % ammonia yielded the title compound (38 mg, 95 %) as colourless oil. ^{1}H-NMR (600 MHz, CD_3OD): δ (ppm) 1.21-1.28 (m, 8H), 1.82 (quint, *J* = 6.8 Hz, 4H), 2.07-2.12 (m, 4H), 2.14 (quint, *J* = 6.8 Hz, 4H), 2.67 (t, *J* = 7.2 Hz, 4H), 2.74 (bs, 8H), 2.90 (t, *J* = 7.4 Hz, 4H), 3.08 (bs, 8H), 3.64 (s, 4H), 3.82 (s, 6H), 4.02 (t, *J* = 6.1 Hz, 4H), 4.11 (t, *J* = 6.2 Hz, 4H), 4.31 (t, *J* = 7.2 Hz, 4H), 6.86-6.88 (m, 2H), 6.91-6.94 (m, 8H), 6.97-7.00 (m, 2H), 7.08 (s, 2H), 7.74 (s, 2H). ^{13}C-NMR (150 MHz, CD_3OD): δ (ppm) 14.5, 22.9, 26.7, 27.3, 29.7, 30.2, 31.1, 51.1, 51.2, 53.9, 56.0, 63.2, 68.6, 69.2, 112.9, 114.9, 117.8, 119.5, 120.9, 122.2, 123.4, 124.7, 124.8, 129.8, 142.0, 148.3, 149.8, 150.3, 153.8. LC-MS-ESI**:** *m/z* Calcd. for $C_{62}H_{82}N_{12}O_6$: 1091.6, $[M+H]^+$: *m/z* Found: 1091.9 $[M+H]^+$, purity LC-MS 96 %, t_R = 16.4 min.

4,4'-(((((Octane-1,8-diylbis(1H-1,2,3-triazole-1,4-diyl))bis(propane-3,1-diyl))bis(oxy))bis(5-((4-(2-methoxyphenyl)piperazin-1-yl)methyl)-2,1-phenylene))bis(oxy))bis(butan-1-amine) (2b)

Compound **10** (29 mg, 0.026 mmol) was dissolved in 2 mL dry THF and cooled down to 0 °C. Then a 4 M solution of $LiAlH_4$ in diethylether (0.07 mL, 0.26 mmol) was added and stirred at 0 °C for 2 hours. It was then quenched with water and filtered through a bed of celite and Na_2SO_4 and washed three times with THF. Preparative HPLC purification employing method D (t_R = 13.5 min), yielded 7 mg (25 %) of **2b** as colourless oil. ^{1}H-NMR (600 MHz, CD_3OD): δ (ppm) 1.26-1.32 (m, 8 H), 1.87 (quint, *J* = 6.9 Hz, 4H), 1.92-1.96 (m, 6H), 2.15-2.18 (m, 4H), 2.91 (bs, 4H), 2.99-3.05 (m, 4H), 3.13 (t, *J* = 7.1 Hz, 4H), 3.24-3.29 (m, 4H), 3.50-3.57 (m, 8H), 3.85 (s, 6H), 4.09-4.12 (m, 6H), 4.32 (bs, 4H), 4.35 (t, *J* = 7.2 Hz, 4H), 6.89-6.92 (m, 2H), 6.95-6.98 (m, 4H), 7.03-7.09 (m, 6H), 7.17-7.19 (m, 2H), 7.85 (bs, 2H). ^{13}C-NMR (150 MHz, CD_3OD): δ (ppm) 23.07, 26.2, 27.4, 29.9, 30.3, 31.2, 31.7, 36.9, 40.8, 51.4, 53.0, 56.0, 61.5, 69.1, 69.9, 101.5, 113.0, 114.5, 117.2, 120.0, 122.2, 122.6, 125.6, 125.9, 140.6, 150.4, 151.6, 154.0,

164.9. LC-MS-ESI**:** *m/z* Calcd. for $C_{62}H_{90}N_{12}O_6$: 550.8, $[M+2H]^{2+}$: *m/z* Found: 550.5 $[M+2H]^{2+}$, purity LC-MS 96 %, t_R = 13.7 min. HRMS Calcd. for $C_{62}H_{91}N_{12}O_6$: 1099.7184, $[M+H]^+$: *m/z* Found: 1099.71791 $[M+H]^+$.

2b-Cy3B conjugate (2c)

Following the GP V, the reaction was performed from compound **2b** (0.6 mg, 0.52 µmol) in DMF (0.3 mL), DIPEA (2.1 µL, 13 µmol) and Cy3B-NHS ester (1 mg, 1.3 µmol), title compound **2c** was prepared. Preparative RP-HPLC by method E, (t_R: 11.2 min) and subsequent lyophilization to afford the conjugate as the TFA salt (1.3 mg, 92 %). Purity and identity was assessed by analytical HPLC coupled to a Bruker Esquire 2000 mass detector equipped with an ESI-trap. Purity: 99% (t_r: 16.2 min), LC-MS-ESI: *m/z* Calcd. for $C_{124}H_{150}N_{16}O_{16}S_2$: 729.3, $[M+3H]^{3+}$: *m/z* Found: 729.7 $[M+3H]^{3+}$.

4-Azidobenzonitrile (11)

Following GP II, from 4-cyanoaniline (4.0 g, 29.1 mmol), 40 mL conc. HCl, $NaNO_2$ (2.00 g, 29.1 mmol) and NaN_3 (18.9 g, 291.7 mmol) 4-azidobenzonitrile was prepared. Yield: 2.8 g (59%). The spectral data were consistent with the previously reported synthesis.

1-(Hex-5-yn-1-yl)-4-(2-(methylthio)phenyl)piperazine (12)

Following GP I, from 1-(2-(methylthio)phenyl)piperazine (0.15 g, 0.7 mmol), 6-chlorohex-1-yne (0.18 mL, 1.4 mmol), K_2CO_3 (0.2 g, 1.4 mmol) and KI (0.12 g, 0.7 mmol) title compound **12** was prepared. It was used for the next step without further purification.

4-(4-(4-(4-(2-(Methylthio)phenyl)piperazin-1-yl)butyl)-1H-1,2,3-triazol-1-yl)benzonitrile (13)

To the mixture of compound **12** (390 mg, 1.35 mmol) and 4-azidobenzonitrile **11** (390 mg, 2.7 mmol) in a solvent system of t-BuOH-H_2O-DCM (1:1:1) was added $CuSO_4.5H_2O$ (17 mg, 0.1mmol) and Na-Ascorbate (28 mg, 0.3 mmol) and was stirred for overnight at room temperature. The reaction was quenched by the addition of H2O. The organic compounds were extracted with DCM (3 x 15 mL), dried over Na_2SO_4 and evaporated. The pure compound was isolated by silica-gel column chromatography pure DCM followed by 95:5 DCM-MeOH to yield 500 mg (86 %) of the pure compound. ^{1}H-NMR (600 MHz, $CDCl_3$): δ (ppm) 1.66-1.71 (m, 2H), 1.81 (quint, *J* = 7.5 Hz, 2H), 2.40 (s, 3H), 2.52 (t, *J* = 7.5 Hz, 2H), 2.69 (bs, 4H), 2.86 (t, *J* = 7.6 Hz, 2H), 3.06 (bs, 4H), 7.04-7.06 (m, 1H), 7.09-7.12 (m, 3H), 7.81-7.83 (m, 2H), 7.87 (s, 1H), 7.90-7.93 (m, 2H).

(4-(4-(4-(4-(2-(Methylthio)phenyl)piperazin-1-yl)butyl)-1H-1,2,3-triazol-1- yl)phenyl)methanamine (14)

Compound **13** (270 mg, 0.62 mmol) was dissolved in 4 mL dry THF and cooled down to 0 °C. Then a 4 M solution of $LiAlH_4$ in diethylether (0.3 mL, 1 mmol) was added and stirred at 0 °C for 2 hours. It was then quenched with water and filtered through a bed of celite and Na_2SO_4 and washed three times with ethylacetate. The solvent was evaporated in vacuo and the crude product was used for the next step.

4-((4-(4-(4-(4-(2-(Methylthio)phenyl)piperazin-1-yl)butyl)-1H-1,2,3-triazol-1-yl)benzyl)amino)-4-oxobutanoic acid (15)

Compound **14** was dissolved in 3 mL DMF in a microwave reaction tube and succinic anhydride and triethyl amine were added. The tube was then sealed and it was heated in microwave for 1h at 100 °C. It was then quenched with water and extracted with ethylacetate three times dried with Na_2SO_4 and the solvent was evaporated in vacuo and the crude product was used for the next step.

Tert-butyl (4-((8-((4-(4-(4-(4-(2-(methylthio)phenyl)piperazin-1-yl)butyl)-1H-1,2,3-triazol-1-yl)benzyl)amino)-5,8-dioxooctyl)amino)butyl)carbamate (16)

To a solution of the compound **15** (100mg, 0.19 mmol) and DIPEA (0.76 mmol, 0.12 ml) in dry DMF, was added a solution of TBTU (0.38 mmol, 121 mg) in DMF at 0 °C. The reaction mixture was stirred for 30 min and then a solution of the free amine (0.22 mmol, 55 mg) in dry DMF was added to the reaction mixture and allowed to stir for overnight at rt. The reaction was quenched by the addition of saturated $NaHCO_3$, extracted with EtOAc (3 x 15 ml), dried over anhydrous Na_2SO_4 and evaporated and the crude product was used for the next step.

N1-(8-Aminooctyl)-N4-(4-(4-(4-(4-(2-(methylthio)phenyl)piperazin-1-yl)butyl)-1H-1,2,3-triazol-1-yl)benzyl)succinamide (17)

To a solution of the Boc compound **16** in DCM, was added 4M HCl in dioxane at room temperature and stirred for overnight. Lyophilization provided the compound as the hydrochloride salt. ^{1}H-NMR (600 MHz, DMSO): δ (ppm) 1.31-1.34 (m, 7H), 1.48 (bs, 2H), 1.66-1.71 (m, 2H), 1.86-1.91 (m, 2H), 2.04-2.09 (m,2H), 2.41 (s, 3H), 2.62 (bs, 4H), 2.82-2.91 (m, 6H), 3.14-3.20 (m, 7H), 3.32 (d, *J* = 13.3 Hz, 2H), 3.50-3.54 (m, 2H), 3.60-3.65 (m, 3H), 4.44 (s, 2H), 7.08-7.17 (m, 4H), 7.46 (d, *J* = 7.9 Hz, 2H), 7.77 (d, *J* = 8.4 Hz, 2H), 8.58 (s, 1H). Purity and identity was assessed by analytical HPLC coupled to a Bruker Esquire 2000 mass detector equipped with an ESI-trap. Purity: 99% (t_r: 18.5 min), LC-MS-ESI: m/z Calcd.

for $C_{36}H_{54}N_8O_2S$: 663.4 $[M+H]^+$: m/z Found: 663.2 $[M+H]^+$, HRMS Calcd. for $C_{36}H_{55}N_8O_2S$: 663.41688 $[M+H]^+$: *m/z* Found: 663.41632 $[M+H]^+$.

17-Cy3B conjugate (4a)

Following the GPV, the reaction was performed from compound **17**·2HCl (1.2 mg, 1.6 µmol) in DMF (0.5 mL), DIPEA (11.6 µmol, 1.8 µL) and Cy3B-NHS ester (1 mg, 1.06 µmol), title compound **4a** was prepared. Preparative RP-HPLC with detection wavelength of 565 nm (eluent: 0.1 % HCO_2H in CH_3CN (A) + H_2O (B) applying a linear gradient 20%-95% A in 80-5 % B in 17 min, t_r: 10.7 min) and subsequent lyophilization to afford the conjugate as the formate salt (2.72 mg). Purity and identity was assessed by analytical HPLC coupled to a Bruker Esquire 2000 mass detector equipped with an ESI-trap. Purity: 99% (t_r: 16.8 min), LC-MS-ESI: m/z Calcd. for $C_{68}H_{85}N_9O_7S$: 587.3, $[M+2H]^{2+}$: m/z Found: 587.1 $[M+2H]^{2+}$.

4-Azidobenzoic acid (18)

Following GP II, from 4-Aminobenzoic acid (4.0 g, 29.1 mmol), 40 mL Conc. HCl, $NaNO_2$ (2.00 g, 29.1 mmol) and NaN_3 (18.9 g, 291.7 mmol) title compound was prepared. **Yield:** 2.8 g (59%). The spectral data were consistent with the previously reported synthesis.

4-(4-(4-(4-(2-(Methylthio)phenyl)piperazin-1-yl)butyl)-1H-1,2,3-triazol-1-yl)benzoic acid (19)

Following GP III, from **12** (63 mg, 0.2 mmol) and 4-azidobenzoic acid **18** (44 mg, 0.27 mmol), title compound **19** was prepared. Flash chromatographic purification with 10-30 % MeOH in ethylacetate gave 81 mg (90 %) of **19** as yellow oil. ^{1}H-NMR (600 MHz, CD_3OD): δ (ppm) 1.67-1.72 (m, 2H), 1.82 (quint, *J* = 7.6 Hz, 2H), 2.39 (s, 3H), 2.56-2.59 (m, 2H), 2.73 (bs, 4H), 2.86 (t, *J* = 7.7 Hz, 2H), 3.03 (bs, 4H), 7.07-7.12 (m 3H), 7.15-7.17 (m, 1H), 7.83-7.85 (m, 2H), 8.12-8.14 (m, 2H), 8.38 (s, 1H). ^{13}C-NMR (150 MHz, CD_3OD): δ (ppm) 14.4, 26.1, 26.8, 28.4, 52.1, 54.6, 59.4, 120.5, 120.7, 121.5, 125.7, 125.8, 126.1, 131.9, 136.4, 139.6, 139.7, 149.9, 150.6, 173.7. LC-MS-ESI**:** *m/z* Calcd. for $C_{24}H_{29}N_5O_2S$: 452.2, $[M+H]^+$: *m/z* Found: 452.7 $[M+H]^+$, purity LC-MS 97 %, t_R = 15.9 min.

N-(3-Aminopropyl)-4-(4-(4-(4-(2-(methylthio)phenyl)piperazin-1-yl)butyl)-1H-1,2,3-triazol-1-yl)benzamide (20)

Following GP IV, from **19** (67 mg, 0.15 mmol), DIPEA (0.05 mL, 0.3mmol), TBTU (72 mg, 0.23 mmol) and 1,3-Diaminopropane (0.13 mL, 1.5 mmol) compound **20** was prepared. Preparative HPLC purification employing method C (t_R = 10 min), yielded 30 mg (40 %) of **20** as colourless oil. ^{1}H-NMR (600 MHz, CD_3OD): δ (ppm) 1.83-1.87 (m, 4H), 1.96-2.01 (m, 2H), 2.41 (s, 3H), 2.89-2.91 (m, 2H), 3.01-3.03 (m, 2H), 3.08-3.10 (m, 2H), 3.20 (bs, 4H), 3.26 (bs, 4H), 3.52 (t, *J* = 7.5 Hz, 2H), 7.11-7.15 (m 3H),

7.18-7.20 (m, 1H), 7.99-8.01 (m 2H), 8.06-8.07 (m, 2H), 8.48 (s, 1H). ^{13}C-NMR (150 MHz, CD_3OD): δ (ppm) 14.3, 25.1, 25.7, 27.5, 28.9, 37.7, 38.4, 50.5, 54.0, 58.2, 120.9, 121.1, 121.7, 125.9, 126.2, 126.6, 130.2, 135.3, 136.6, 140.8, 149.2, 169.5. LC-MS-APCI**:** *m/z* Calcd. for $C_{27}H_{37}N_7OS$: 508.3, $[M+H]^+$: *m/z* Found: 508.9 $[M+H]^+$, purity LC-MS 98 %, t_R = 12.5 min, HRMS Calcd. for $C_{27}H_{38}N_7OS$: 508.28491, $[M+H]^+$: *m/z* Found: 508.28531 $[M+H]^+$.

20-Cy3B conjugate (4b)

Following the GP V, the reaction was performed from compound **20** (1.4 mg, 2.7 µmol) in DMF (0.5 mL), DIPEA (2.3 µL, 13 µmol) and Cy3B-NHS ester (0.4 mg, 0.5 µmol), title compound **4b** was prepared. Preparative RP-HPLC by method B, (t_R: 17.7 min) and subsequent lyophilization to afford the conjugate as the TFA salt (0.6 mg, 89 %). Purity and identity was assessed by analytical HPLC coupled to a Bruker Esquire 2000 mass detector equipped with an ESI-trap. Purity: 97% (t_r: 16.0 min), LC-MS-ESI: *m/z* Calcd. for $C_{58}H_{68}N_9O_6S_2$: 526.5, $[M+2H]^{2+}$: *m/z* Found: 526.5 $[M+2H]^2$

(S)-N-(Hex-5-yn-1-yl)-5-methoxy-N-propyl-1,2,3,4-tetrahydronaphthalen-2-amine (21)

Following GP I, from (S)-1,2,3,4-Tetrahydro-5-methoxy-N-propyl-2-naphthalenamine hydrochloride (0.1 g, 0.4 mmol), 6-chlorohex-1-yne (0.05 mL, 0.4 mmol), K_2CO_3 (0.112 g, 0.8 mmol) and KI (67 mg, 0.4 mmol) title compound **2** was prepared. It was used for the next step without further purification.

(S)-4-(4-(4-((5-Methoxy-1,2,3,4-tetrahydronaphthalen-2-yl)(propyl)amino)butyl)-1H-1,2,3-triazol-1-yl)benzonitrile (22)

Following GP III, **21** (83 mg, 0.3 mmol) and 4-azidobenzonitrile **11** (109mg, 0.7 mmol), title compound **22** was prepared. Flash chromatographic purification with 5 % MeOH in ethylacetate gave 75 mg (62 %) of **22** as yellow oil. ^{1}H-NMR (600 MHz, $CDCl_3$): δ (ppm) 0.90 (t, *J* = 7.4 Hz, 3H), 1.52 (sextet, *J* = 7.2 Hz, 2H), 1.56-1.65 (m, 3H), 1.79 (quint, *J* = 7.5 Hz, 2H), 2.07-2.11 (m, 1H), 2.48-2.57 (m, 3H), 2.62-2.67 (m, 2H), 2.76-2.81 (m, 1H), 2.83-2.88 (m 3H), 2.97-3.03 (m, 2H), 3.8 (s, 3H), 6.65 (d, *J* = 8.4 Hz, 1H), 6.70 (d, *J* = 7.7 HZ, 1H), 7.08 (t, *J* = 8.0 HZ, 1H), 7.80-7.82 (m, 2H), 7.87 (s, 1H), 7.90-7.92 (m, 2H). ^{13}C-NMR (150 MHz, CD_3OD): δ (ppm) 11.9, 21.6, 23.8, 25.3, 25.4, 26.9, 27.9, 31.8, 50.2, 52.4, 55.2, 56.6, 107.0, 111.9, 117.8, 118.7, 120.3, 121.6, 125.0, 126.3, 113.8, 137.4, 139.9, 149.6, 157.2. LC-MS-APCI**:** *m/z* Calcd. for $C_{27}H_{33}N_5O$: 444.2, $[M+H]^+$: *m/z* Found: 444.6 $[M+H]^+$, purity LC-MS 98 %, t_R = 16.7 min. $[\alpha]_{589}$ = -28.9° (22 °C, c = 1.32, MeOH).

(S)-N-(4-(1-(4-(Aminomethyl)phenyl)-1H-1,2,3-triazol-4-yl)butyl)-5-methoxy-N-propyl-1,2,3,4-tetrahydronaphthalen-2-amine (23)

Compound **22** (29 mg, 0.065 mmol) was dissolved in 2 mL dry THF and cooled down to 0 °C. Then a 4 M solution of $LiAlH_4$ in diethylether (0.07 mL, 0.26 mmol) was added and stirred at 0 °C for 2 hours. It was then quenched with water and filtered through a bed of celite and Na_2SO_4 and washed three times with ethylacetate. The solvent was evaporated in vacuo and the crude product was used for the next step.

(S)-4-((4-(4-(4-((5-Methoxy-1,2,3,4-tetrahydronaphthalen-2-yl)(propyl)amino)butyl)-1H-1,2,3-triazol-1-yl)benzyl)amino)-4-oxobutanoic acid (24)

Compound **23** was dissolved in 3 mL DMF in a microwave reaction tube and succinic anhydride (32 mg, 0.33 mmol) and triethyl amine (0.05 mL, 0.33 mmol) were added. The tube was then sealed and it was heated in microwave for 1h at 100 °C. It was then quenched with water and extracted with ethylacetate three times dried with Na_2SO_4 and the solvent was evaporated in vacuo. Flash chromatographic purification using 30 % methanol in ethylacetate yielded 27 mg (75 %) of **24** as yellow oil. ^{1}H-NMR (360 MHz, CD_3OD): δ (ppm) 0.95 (t, *J* = 7.4 Hz, 3H), 1.55-1.64 (m, 2H), 1.66-1.73 (m, 2H), 1.82 (quint, *J* = 7.2 Hz, 2H), 2.13-2.20 (m, 1H), 2.50-2.57 (m, 7H), 2.73-2.78 (m, 2H), 2.83-2.88 (m, 4H), 2.91-3.04 (m, 2H), 3.12-3.20 (m, 1H), 3.80 (s, 3H), 4.45 (s, 2H), 6.70 (t, *J* = 8.5 Hz, 2H), 7.07 (t, *J* = 8.0 Hz, 1H), 7.48-7.52 (m, 2H), 7.75-7.79 (m, 2H), 8.31 (s, 1H). ^{13}C-NMR (150 MHz, CD_3OD): δ (ppm) 11.9, 21.6, 24.4, 25.9, 26.0, 27.6, 28.0, 32.3, 34.0, 43.5, 51.5, 53.7, 55.7, 59.2, 108.4, 121.4, 121.5, 122.5, 125.5, 127.7, 129.8, 129.9, 137.4, 141.4, 149.7, 158.7, 176.0. LC-MS-APCI**:** *m/z* Calcd. for $C_{31}H_{41}N_5O_4$: 548.3, $[M+H]^+$: *m/z* Found: 548.9 $[M+H]^+$, purity LC-MS 98 %, t_R = 16.1 min. $[\alpha]_{589}$ = -17.5° (24 °C, c = 0.64, MeOH).

(S)-N1-(8-Aminooctyl)-N4-(4-(4-(4-((5-methoxy-1,2,3,4-tetrahydronaphthalen-2-yl)(propyl)amino)butyl)-1H-1,2,3-triazol-1-yl)benzyl)succinamide (25)

Following GP IV, from **24** (53 mg, 0.095 mmol), DIPEA (0.03 mL, 0.2 mmol), TBTU (46 mg, 0.14 mmol) and 1,8-Diaminooctane (0.15 mL, 0.95 mmol) compound **25** was prepared. Flash chromatographic purification using 30 % Methanol in ethylacetate and 1 % NH_3 solution yielded 28 mg (40 %) of **25** as colourless oil. ^{1}H-NMR (600 MHz, CD_3OD): δ (ppm) 0.89 (t, *J* = 7.3 Hz, 3H), 1.44-1.53 (m, 13H), 1.54-1.60 (m, 2H), 1.77 (quint, *J* = 7.5 Hz, 2H), 2.04-2.08 (m, 1H), 2.42-2.48 (m, 1H), 2.49-2.57 (m, 6H), 2.60-2.68 (m 6H), 2.71 (dd, *J* = 15.7, 11.6 Hz, 1H), 2.80-2.83 (m, 3H), 2.86-2.91 (m, 1H), 2.95 (ddd, *J* = 17.7, 5.7, 2.3 Hz, 1H), 3.14 (t, *J* = 7.4 Hz, 2H), 3.20 (t, *J* = 7.4 Hz, 2H), 3.77 (s, 3H), 4.43 (s, 2H), 6.64 (d, *J* = 7.7

Hz, 1H), 6.66 (d, *J* = 7.7 Hz, 1H), 7.02 (t, *J* = 7.7 Hz, 1H), 7.46-7.48 (m, 2H), 7.75-7.77 (m, 2H), 8.01 (s, 1H), 8.28 (s, 1H). ^{13}C-NMR (150 MHz, CD_3OD): δ (ppm) 12.2, 22.5, 24.7, 26.1, 26.6, 27.8, 27.9, 28.3, 28.7, 30.2, 30.3, 30.4, 32.2, 32.3, 33.0, 38.9, 40.5, 42.3, 43.5, 51.3, 53.7, 55.7, 58.0, 108.1, 121.4, 122.6, 125.9, 127.3, 129.9, 137.4, 138.4, 141.1, 149.9, 158.5, 163.7, 174.4, 174.7. LC-MS-ESI**:** *m/z* Calcd. for $C_{39}H_{59}N_7O_3$: 674.5, $[M+H]^+$: *m/z* Found: 675.2 $[M+H]^+$, purity LC-MS 98 %, t_R = 15.1 min. $[\alpha]_{589}$ = -15.1° (20 °C, c = 1.03, MeOH).

(S)-N1-(8-Aminooctyl)-N4-(4-(4-(4-((5-hydroxy-1,2,3,4-tetrahydronaphthalen-2-yl)(propyl)amino)butyl)-1H-1,2,3-triazol-1-yl)benzyl)succinamide (26)

Compound **25** (15 mg, 0.02 mmol) was dissolved in 2 mL of DCM and cooled down to -40 °C. Then a solution of BBr_3 (1M in DCM, 0.07 mL, 0.06 mmol) was added and stirred at -40 °C for 2 hours. It was then warmed up to room temperature and stirred overnight at room temperature. The reaction was quenched with satd. $NaHCO_3$ solution and extracted three times with DCM. The organic layer was collected and dried with Na_2SO_4 and concentrated in vacuo. Preparative HPLC purification employing method A (t_R = 15 min), yielded 8 mg (52 %) of **26** as colourless oil. ^{1}H-NMR (600 MHz, CD_3OD): δ (ppm) 1.04 (t, *J* = 7.3 Hz, 3H), 1.31-1.39 (m, 9H), 1.49 (quint, *J* = 7.2 Hz, 2H), 1.63 (m, *J* = 7.3 Hz, 2H), 1.75-1.91 (m, 8H), 2.30-2.33 (m, 1H), 2.49-2.52 (m, 2H), 2.55-2.57 (m, 2H), 2.61-2.67 (m, 1H), 2.89-2.91 (m, 4H), 3.04-3.10 (m, 3H), 3.12-3.17 (m, 3H), 3.20-3.29 (m, 2H), 3.34-3.39 (m 1H), 3.68-3.73 (m, 1H), 4.44 (s, 2H), 6.62 (t, *J* = 7.9 Hz, 2H), 6.96 (t, *J* = 7.6 Hz, 1H), 7.48 (d, *J* = 8.3 Hz, 2H), 7.77 (d, *J* = 8.0 Hz, 2H), 8.37 (s, 1H). ^{13}C-NMR (150 MHz, CD_3OD): δ (ppm) 11.3, 19.7, 22.6, 23.7, 24.9, 25.4, 25.5, 27.4, 27.8, 28.6, 30.1, 30.4, 30.7, 30.8, 32.1, 32.2, 40.4, 40.8. 43.5, 52.2, 54.0, 61.7, 113.5, 121.2, 121.5, 123.1, 128.1, 129.9, 134.8, 137.4, 141.3, 156.2, 174.5, 174.8. LC-MS-ESI**:** *m/z* Calcd. for $C_{38}H_{57}N_7O_3$: 660.5, $[M+H]^+$: *m/z* Found: 661.0 $[M+H]^+$, purity LC-MS 98 %, t_R = 14.2 min. $[\alpha]_{589}$ = -19.1° (22 °C, c = 0.5, MeOH). HRMS Calcd. for $C_{38}H_{58}N_7O_3$: 660.46091, $[M+H]^+$: *m/z* Found: 660.45957 $[M+H]^+$.

26-Cy3B conjugate (5a)

Following the GP V, the reaction was performed from compound **26** (0.9 mg, 1.3 µmol) in DMF (0.5 mL), DIPEA (1.2 µL, 6.5 µmol) and Cy3B-NHS ester (0.5 mg, 0.65 µmol), title compound **5a** was prepared. Preparative RP-HPLC by method B, (t_R: 17.9 min) and subsequent lyophilization to afford the conjugate as the TFA salt (0.8 mg, 92 %). Purity and identity was assessed by analytical HPLC coupled to a Bruker Esquire 2000 mass detector equipped with an ESI-trap. Purity: 99% (t_r: 16.2 min), LC-MS-ESI: *m/z* Calcd. for $C_{69}H_{87}N_9O_8S$: 602.3, $[M+2H]^{2+}$: *m/z* Found: 602.5 $[M+2H]^{2+}$.

(S)-4-(4-(4-((5-Methoxy-1,2,3,4-tetrahydronaphthalen-2-yl)(propyl)amino)butyl)-1H-1,2,3-triazol-1-yl)benzoic acid (27)

Following GP III, from **21** (121 mg, 0.4 mmol) and 4-azidobenzoic acid **18** (44 mg, 0.27 mmol), title compound **27** was prepared. Flash chromatographic purification with 10-30 % MeOH in ethylacetate gave 74 mg (60 %) of **27** as yellow oil. ^{1}H-NMR (600 MHz, DMSO D6): δ (ppm) 0.82 (t, *J* = 7.2 Hz, 3H), 1.38 (sextet, *J* = 7.2 Hz, 2H), 1.44-1.51 (m, 3H), 1.69 (quintet, *J* = 7.4 Hz, 2H), 1.90-1.93 (m, 1H), 2.38-2.44 (m, 3H), 2.51-2.53 (m,2H), 2.63-2.67 (m, 1H), 2.69-2.73 (m, 3H), 2.77-2.86 (m, 2H), 3.72 (s, 3H), 6.65 (d, *J* = 7.5 Hz, 1H), 6.69 (d, *J* = 8.0 Hz, 1H), 7.02 (t, *J* = 7.9 Hz, 1H), 7.80 (d, *J* = 8.6 Hz, 2H), 8.04 (d, *J* = 8.6 Hz, 2H), 8.56 (s, 1H). ^{13}C-NMR (90 MHz, DMSO D6): δ (ppm) 11.6, 21.6, 23.4, 24.8, 24.9, 26.6, 27.8, 31.7, 49.4, 51.7, 55.0, 55.5, 107.0, 118.5, 119.9, 121.3, 124.3,126.0, 130.4, 137.3, 137.6, 148.1, 156.7, 168.2. LC-MS-ESI**:** *m/z* Calcd. for $C_{27}H_{34}N_4O_3$: 463.2, $[M+H]^+$: *m/z* Found: 463.4 $[M+H]^+$, purity LC-MS 98 %, t_R = 16.2 min, $[\alpha]_{589}$ = -25.6° (26 °C, c = 1.62, MeOH).

(S)-N-(3-Aminopropyl)-4-(4-(4-((5-methoxy-1,2,3,4-tetrahydronaphthalen-2-yl)(propyl)amino)butyl)-1H-1,2,3-triazol-1-yl)benzamide (28)

Following GP IV, from **27** (21 mg, 0.045 mmol), DIPEA (0.015 mL, 0.09 mmol), TBTU (21 mg, 0.07 mmol) and 1,3-Diaminopropane (0.04 mL, 0.45 mmol) compound **28** was prepared. Flash chromatographic purification with 20-50 % MeOH in ethyl acetate and 0.1 % diethyl amine yielded 21 mg (90 %) of **28** as yellow oil. ^{1}H-NMR (600 MHz, CD_3OD): δ (ppm) 0.89 (t, *J* = 7.3 Hz, 3H), 1.46-1.60 (m, 6H), 1.75-1.81 (m, 4H), 2.03-2.07 (m, 1H), 2.41-2.47 (m, 1H), 2.50-2.53 (m, 2H), 2.60-2.63 (m, 2H), 2.65-2.75 (m, 3H), 2.82 (t, *J* = 7.5 Hz, 2H), 2.85-2.90 (m, 1H), 2.94 (ddd, *J* = 17.5, 5.6, 2.1 Hz, 1H), 3.48 (t, *J* = 6.7 Hz, 2H), 3.76 (s, 3H), 6.63 (d, *J* = 7.5 Hz, 1H), 6.65 (d, *J* = 8.0 Hz, 1H), 7.01 (t, *J* = 8.0 Hz, 1H), 7.92-7.94 (m, 2H), 7.99-8.01 (m, 2H), 8.39 (s, 1H). ^{13}C-NMR (90 MHz, CD_3OD): δ (ppm) 12.2, 22.5, 24.8, 26.2, 26.6, 28.3, 28.7, 33.1, 33.4, 38.5, 39.9, 51.3, 53.8, 55.7, 58.1, 108.1, 121.0, 121.4, 122.6, 125.9, 127.4, 130.1, 135.8, 138.5, 140.6, 150.3, 158.6, 168.9. LC-MS-ESI**:** *m/z* Calcd. for $C_{30}H_{42}N_6O_2$: 519.3, $[M+H]^+$: *m/z* Found: 519.8 $[M+H]^+$, purity LC-MS 95 %, t_R = 13.0 min, $[\alpha]_{589}$ = -20.3° (25 °C, c = 0.96, MeOH).

(S)-N-(3-Aminopropyl)-4-(4-(4-((5-hydroxy-1,2,3,4-tetrahydronaphthalen-2-yl)(propyl)amino)butyl)-1H-1,2,3-triazol-1-yl)benzamide (29)

Compound **28** (20 mg, 0.04 mmol) was dissolved in 2 mL of DCM and cooled down to -40 °C. Then a solution of BBr_3 (1M in DCM, 0.1 mL, 0.1 mmol) was added and stirred at -40 °C for 2 hours. It was then warmed up to room temperature and stirred overnight at room temperature. The reaction was quenched with satd. $NaHCO_3$ solution and extracted three times with DCM. The organic layer was collected and dried with Na_2SO_4 and concentrated in vacuo. Preparative HPLC purification employing

method A (t_R = 13 min), yielded 17 mg (84 %) of **29** as colourless oil. ^{1}H-NMR (600 MHz, CD_3OD): δ (ppm) 1.03 (t, *J* = 7.4 Hz, 3H), 1.73-1.92 (m, 7H), 1.95-2.02 (m, 2H), 2.27-2.33 (m, 1H), 2.57-2.67 (m, 1H), 2.88-2.91 (m, 2H), 2.99-3.12 (m, 5H), 3.16-3.21 (m, 2H), 3.24-3.29 (m, 2H), 3.53 (t, *J* = 6.8 Hz, 2H), 3.64-3.71 (m, 1H), 6.62 (t, *J* = 6.7 Hz, 2H), 6.94 (t, *J* = 7.8 Hz, 1H), 7.97 (d, *J* = 8.8 Hz, 2H), 8.05 (d, *J* = 8.8 Hz, 2H), 8.45 (s, 1H). ^{13}C-NMR (90 MHz, CD_3OD): δ (ppm) 11.3, 19.8, 23.7, 24.9, 25.5, 25.6, 27.3, 28.9, 30.8, 37.6, 38.4, 51.9, 53.8, 61.6, 113.5, 121.0, 121.2, 121.6, 123.1, 127.9, 130.2, 134.9, 135.3, 140.7, 149.5, 156.1, 169.4. LC-MS-ESI**:** *m/z* Calcd. for $C_{29}H_{40}N_6O_2$: 505.3, $[M+H]^+$: *m/z* Found: 505.8 $[M+H]^+$, purity LC-MS 99 %, t_R = 12.3 min, $[\alpha]_{589}$ = -21.3° (23 °C, c = 1.26, MeOH). HRMS Calcd. for $C_{29}H_{41}N_6O_2$: 505.32847, $[M+H]^+$: *m/z* Found: 505.32855 $[M+H]^+$.

29-Cy3B conjugate (5b)

Following the GP V, the reaction was performed from compound **29** (0.8 mg, 1.6 µmol) in DMF (0.5 mL), DIPEA (8 µmol, 1.4 µL) and Cy3B-NHS ester (0.6 mg, 0.8 µmol), title compound **5b** was prepared. Preparative RP-HPLC by method B, (t_R: 17 min) and subsequent lyophilization to afford the conjugate as the TFA salt (0.75 mg, 89 %). Purity and identity was assessed by analytical HPLC coupled to a Bruker Esquire 2000 mass detector equipped with an ESI-trap. Purity: 99% (t_r: 15.7 min), LC-MS-ESI: *m/z* Calcd. for $C_{60}H_{71}N_8O_7S$: 524.5, $[M+2H]^{2+}$: *m/z* Found: 524.8 $[M+2H]^{2+}$.

Supplementary Movies

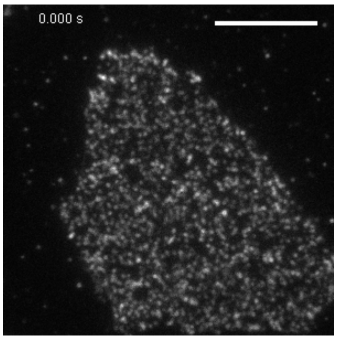

Supplementary Movie S1. First 100 frames of a TIRF-M recording of the diffusion of individual SNAP-D_{2L} receptors labeled with Alexa546-BG on the plasma membrane of a single CHO cell (50 ms exposure time, frame rate of 19.32 fps). An image of this cell is shown in Figure 1b (main text).

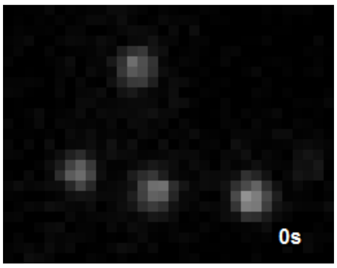

Supplementary Movie S2. Single fluorescent spots of Alexa546-BG fluorophores showed photobleaching during laser exposure (50 ms exposure time, frame rate of 19.32 fps).

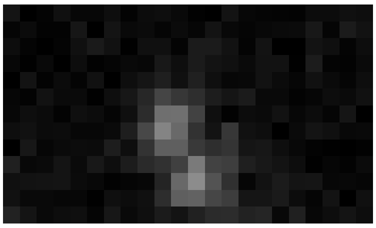

Supplementary Movie S3. Transient dimer formation of two Alexa546-labeled SNAP-D_{2L} receptors (50 ms exposure time, frame rate of 19.32 fps).

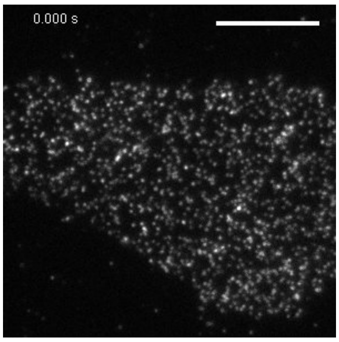

Supplementary Movie S4. First 100 frames of a TIRF-M recording of the diffusion of individual D_{2L} receptors labeled the fluorescent antagonist **1c** on the plasma membrane of a single CHO cell (50 ms exposure time, frame rate of 19.32 fps). An image of this cell is shown in Figure 7e (main text).

Supplementary References

1. Jones KA, *et al.* GABAB receptors function as a heteromeric assembly of the subunits GABABR1 and GABABR2. *Nature* **396**, 674-679 (1998).

2. Huang J, Chen S, Zhang JJ, Huang X-Y. Crystal structure of oligomeric β1-adrenergic G protein-coupled receptors in ligand-free basal state. *Nat Struct Mol Biol* **20**, 419-425 (2013).

3. Crivat G, Taraska JW. Imaging proteins inside cells with fluorescent tags. *Trends in Biotechnology* **30**, 8-16.

4. Kruse AC, *et al.* Activation and allosteric modulation of a muscarinic acetylcholine receptor. *Nature* **504**, 101-106 (2013).
